# Bacteria are important dimethylsulfoniopropionate producers in marine aphotic and high-pressure environments

**DOI:** 10.1038/s41467-020-18434-4

**Published:** 2020-09-16

**Authors:** Yanfen Zheng, Jinyan Wang, Shun Zhou, Yunhui Zhang, Ji Liu, Chun-Xu Xue, Beth T. Williams, Xiuxiu Zhao, Li Zhao, Xiao-Yu Zhu, Chuang Sun, Hong-Hai Zhang, Tian Xiao, Gui-Peng Yang, Jonathan D. Todd, Xiao-Hua Zhang

**Affiliations:** 1 College of Marine Life Sciences, and Institute of Evolution & Marine Biodiversity, Ocean University of China, Qingdao, 266003 China; 2 School of Biological Sciences, University of East Anglia, Norwich, NR4 7TJ UK; 3 Key Laboratory of Marine Ecology and Environmental Sciences, Institute of Oceanology, Chinese Academy of Sciences, Qingdao, 266071 China; 4 MOE Key Laboratory of Marine Chemistry Theory and Technology, College of Chemistry and Chemical Engineering, Ocean University of China, Qingdao, 266100 China; 5 Laboratory for Marine Ecology and Environmental Science, Qingdao National Laboratory for Marine Science and Technology, Qingdao, 266237 China

**Keywords:** Water microbiology, Element cycles, Microbial ecology

## Abstract

Dimethylsulfoniopropionate (DMSP) is an important marine osmolyte. Aphotic environments are only recently being considered as potential contributors to global DMSP production. Here, our Mariana Trench study reveals a typical seawater DMSP/dimethylsulfide (DMS) profile, with highest concentrations in the euphotic zone and decreased but consistent levels below. The genetic potential for bacterial DMSP synthesis via the *dsyB* gene and its transcription is greater in the deep ocean, and is highest in the sediment.s DMSP catabolic potential is present throughout the trench waters, but is less prominent below 8000 m, perhaps indicating a preference to store DMSP in the deep for stress protection. Deep ocean bacterial isolates show enhanced DMSP production under increased hydrostatic pressure. Furthermore, bacterial *dsyB* mutants are less tolerant of deep ocean pressures than wild-type strains. Thus, we propose a physiological function for DMSP in hydrostatic pressure protection, and that bacteria are key DMSP producers in deep seawater and sediment.

## Abstract (web summary)

Dimethylsulfoniopropionate (DMSP) is an osmolyte produced by marine microbes that plays an important role in nutrient cycling and atmospheric chemistry. Here the authors go to the Mariana Trench—the deepest point in the ocean—and find bacteria are key DMSP producers, and that DMSP has a role in protection against high pressure.

## Introduction

Petagrams of dimethylsulfoniopropionate (DMSP) are produced annually^1^ by marine algae, corals, plants, and heterotrophic bacteria^2^. In these organisms, DMSP is proposed to act as an osmolyte, cryoprotectant, predator deterrent, and/or antioxidant^3–5^. DMSP is synthesized from methionine (Met) via three distinct synthesis pathways (Supplementary Fig. 1)^3–5^. The homologous *dsyB* (in bacteria) and *DSYB* (in algae) genes, and *TpMMT* in the diatom *Thalassiosira pseudonana* encode the key methylthiohydroxybutyrate methyltransferase enzyme of the transamination pathway^6–8^. In bacteria producing DMSP via the Met methylation pathway, *mmtN* encodes the key Met methyltransferase enzyme^9,10^. Recent studies suggest bacteria are likely important DMSP producers in coastal sediments, which have far higher DMSP-standing stocks than surface seawater samples where phytoplankton likely drive DMSP production^7,10^.

DMSP, released into the environment through grazing and/or virus-induced lysis, provides a key source of carbon, reduced sulfur, and/or energy for microbial communities^11,12^. Many bacteria and phytoplankton catabolize DMSP via diverse DMSP lyase enzymes to generate the climate-active volatile dimethylsulfide (DMS)^2,11^. DMS is an info-chemical^13,14^ and the largest biogenic source of atmospheric sulfur, with roles in cloud formation and, potentially, climate regulation^15,16^. Alternatively, bacteria with the DMSP demethylase enzyme (DmdA) demethylate DMSP, which is thought to be quantitatively more important than lysis^17,18^.

Seawater DMSP levels in the photic zone (above 200 m) vary from 1 to 100 nM in the oligotrophic ocean^19–23^ to micromolar levels in phytoplankton blooms^24,25^, and are generally highest in chlorophyll *a* (Chl-*a*) maximum layers^21^. Marine aphotic seawaters (below 200 m) have lower DMSP levels (~1.0–3.3 nM) in comparison^26,27^, but represent a much larger global volume. There are few analyses of DMSP in deep-ocean sediment and seawater^2^, and none investigating bacterial DMSP production and cycling. Recently, a 4500 m deep Mariana Trench sediment sample was shown to have high *dsyB* transcript levels and far higher DMSP levels than in surface water samples^10^, highlighting the need for further surveys of deep ocean organosulfur cycling.

**Fig. 1:**
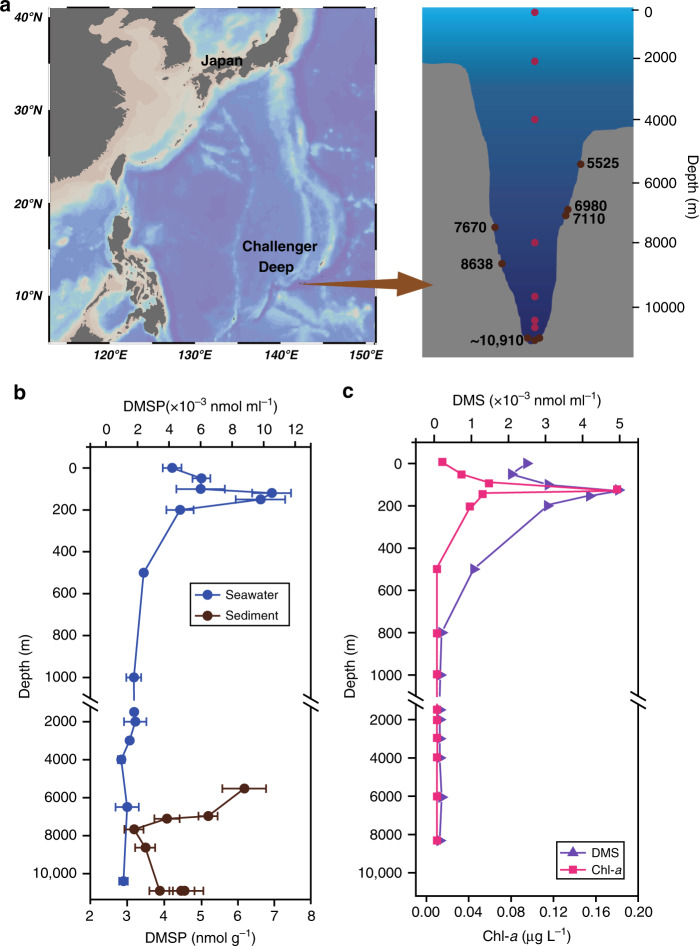
Depth profile of DMSP, DMS, and chlorophyll *a* in Challenger Deep seawater and sediment samples. **a** Sampling sites and depths. **b** Total DMSP concentrations in seawater and sediment samples. **c** Total DMS and chlorophyll *a* in seawater samples. Data in **b** are presented as means ± SD.

Following the hypothesis that bacteria are important DMSP producers in marine aphotic environments, the microbes synthesizing and catabolizing DMSP were examined in seawater and sediment samples (surface water—10,500 m depths) from the Challenger Deep of the Mariana Trench (Fig. 1). The DMSP and DMS stocks were determined in depth-profiled seawater and sediment samples, together with bacterial DMSP synthesis, catabolic gene, and transcript abundance by metagenomics and quantitative PCR (qPCR) analyses. Furthermore, deep ocean bacteria were isolated and used to explore a role for DMSP in hydrostatic pressure tolerance. This work provides insights into the bacterial contribution to DMSP production and function in the deep ocean.

## Results

### Environmental parameters of deep ocean seawater and sediment

Challenger Deep seawater and surface sediment samples were taken from its entire ~11,000 depth profile (Fig. 1a and Supplementary Table 1). The clines in temperature (29.8 °C in surface waters, decreasing to ~1.0 °C below 3000 m) and pressure (0.1 MPa in surface waters to ~104 MPa at the bottom of the trench) were recorded. The waters were oxic throughout the water column and the salinity ranged between 34 and 35 Practical Salinity Units (PSUs) (Supplementary Table 1). Seawater total DMSP and DMS concentrations were similar to those in previous studies^21,26–28^ and were positively correlated with Chl-*a* levels, being highest in the Chl-*a* maximum layer (10.51 × 10^−3^ nmol ml^−1^ DMSP and 4.97 × 10^−3^ nmol ml^−1^ DMS) and at lower but relatively stable levels (0.96–2.39 × 10^−3^ nmol ml^−1^ DMSP and 0.15–1.06 × 10^−3^ nmol ml^−1^ DMS) in the aphotic waters below 200 m (Fig. 1b, c and Supplementary Table 1). It should be noted that a small portion of this ‘background DMSP’ (<1 nM) detected through alkaline hydrolysis may arise from other organic sulfur compounds that also release DMS upon chemical lysis^29^. Heterotrophic bacteria, photosynthetic phytoplankton (~1.5% of the total microbial community data determined by metagenomics and 16S rRNA gene amplicon analyses), picoeukaryotes, *Prochlorococcus*, and *Synechococcus* were most abundant in the surface waters with the highest seawater DMSP concentration (Supplementary Figs. 2 and 3). Bacteria were present consistently at 10^5^ cells ml^−1^ levels throughout the water column, whereas the phototrophs were barely detected below the first 200 m (Supplementary Fig. 2). Surface water cyanobacteria likely take up DMSP^30^ but are unlikely to be significant DMSP producers or cyclers, as few are proven to synthesize it (and at very low levels)^31^ and none contain known DMSP synthesis or catabolic genes. Dinoflagellates of the *Dinophysis* genus, e.g., *Dinophysis* *acuminata* that contains *DSYB*, has intracellular DMSP levels of 477 mM and large cells (30–120 µm)^32^, and were the most abundant surface water phytoplankton (up to 73% of detected phytoplankton) (Supplementary Fig. 3). These phytoplankton were likely the major contributors to the DMSP levels detected in the photic waters, although Picoeukaryotes, proposed to contain *DSYB*^7^, and DMSP-producing bacteria (see below) likely also contributed. However, no eukaryotic DMSP synthesis genes (*DSYB* or *TpMMT*) were detected in any metagenomes (Table 1), even from the surface waters, perhaps reflecting the need for deeper sequencing of these waters where phytoplankton are far less abundant than bacteria.

**Table 1:** Metagenome information and results of DMSP-related gene searches in the metagenome data.

Samples	Depth (m)	Estimated % of cell with												
		DMSP biosynthesis				DMSP cleavage								DMSP demethylation
		DsyB	MmtN	DSYB	TpMMT	DddP	DddQ	DddL	DddD	DddK	DddW	DddY	Alma1	DmdA
FL0^a^	0	0.78 ± 0.16	0.40 ± 0.18	0	0	6.48 ± 0.24	0.96 ± 0.14	0.64 ± 0.06	0.35 ± 0.16	0.26 ± 0.07	0.00	0.01^c^	0	35.73 ± 7.96
PA0^a^	0	0.89 ± 0.17	0.23 ± 0.01	0	0	6.05 ± 1.43	0.24 ± 0.17	1.29 ± 0.56	0.33 ± 0.05	0.29^c^	0.09^c^	0.00	0	12.73 ± 0.89
FL2000^b^	2000	0.43 ± 0.15	0.47 ± 0.16	0	0	5.34 ± 0.20	3.55 ± 0.63	1.09 ± 0.14	0.10 ± 0.06	0.00	0.00	0.13 ± 0.06	0	26.66 ± 7.17
FL4000^a^	4000	3.28 ± 1.88	0.33 ± 0.12	0	0	2.74 ± 0.87	1.79 ± 0.64	3.03 ± 0.41	0.24 ± 0.04	0.00	0.35^c^	0.12^c^	0	6.01 ± 0.99
PA4000^a^	4000	2.18 ± 0.02	0.65 ± 0.55	0	0	5.29 ± 1.85	0.35 ± 0.14	4.61 ± 1.91	0.34 ± 0.17	0.00	0.15 ± 0.05	0.03^c^	0	5.43 ± 3.16
FL8000^b^	8000	4.03 ± 1.92	1.22 ± 0.24	0	0	6.00 ± 0.61	0.71 ± 0.11	4.00 ± 1.19	1.61 ± 0.12	0.03^c^	0.00	0.06^c^	0	10.37 ± 0.88
FL9600	9600	2.23	0.36	0	0	1.03	0	1.40	0.19	0.00	0.00	0.00	0	1.65
FL10400	10,400	2.63	0.28	0	0	0.00	0	0.00	0.14	0.00	0.00	0.00	0	0.00
PA10400	10,400	2.26	0.00	0	0	0.24	0	0.64	0.30	0.00	0.00	0.00	0	0.00
FL10500	10,500	3.53	0.57	0	0	0.35	0.07	0.51	0.14	0.00	0.13	0.00	0	0.70
PA10500	10,500	3.58	0.28	0	0	0.12	0	0.42	0.34	0.00	0.12	0.00	0	0.42

*FL* free-living, *PA* particle-associated. Bacterial sequences were normalized to gene length. Estimated percentages were calculated using *recA* abundance. Data shown are means ± SD.

^a^Mean of two replicates, which are from different cruises but the same station (September 2016 and March 2017).

^b^Mean of two replicates, which are from the same cruise (March 2017).

^c^Values from one cruise when another value is zero.

The deep ocean surface sediment DMSP concentrations (3.15–6.14 nmol g^−1^) were two to three orders of magnitude higher than in the corresponding seawater samples per equivalent mass (ml vs. g) (Fig. 1c and Supplementary Table 1), consistent with previous observations of coastal sediments^10,33^. Given the cold, dark, and high-pressure deep-sediment and -water conditions where few live phytoplankton are present (Supplementary Figs. 2 and 3), it is unlikely these phototrophs produced the observed aphotic DMSP in situ, although some is expected to arise from sinking particles, e.g., dead algae and/or fecal pellets^34,35^. However, considering the high DMSP turnover rates in photic seawater samples^4,36^, it is unlikely that photic-produced DMSP is the source of all aphotic DMSP. We propose that bacterial DMSP synthesis is likely an important contributor to deep-sea sediment and seawater DMSP levels. To test this hypothesis, the distribution and activity of DMSP-producing bacteria was investigated in Challenger Deep samples.

### Vertical distribution of DMSP synthesis genes

**Fig. 2:**
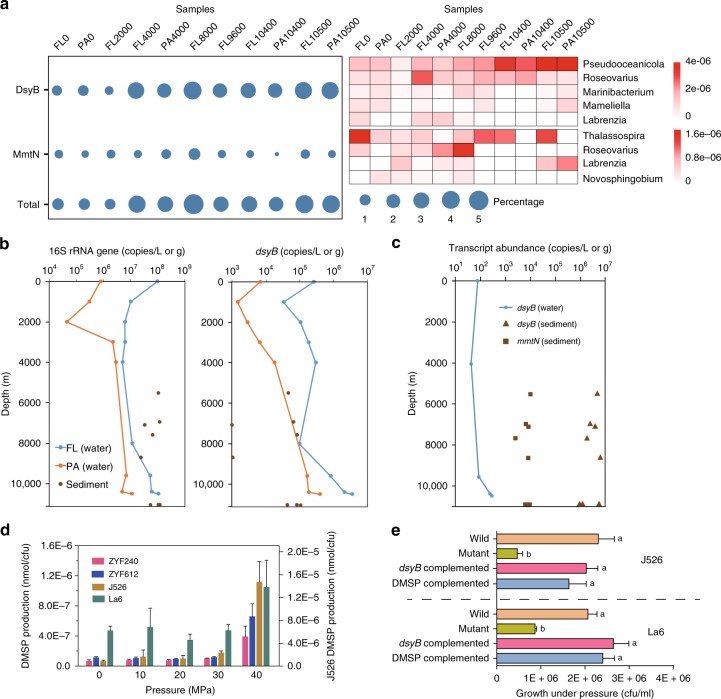
The importance of DMSP synthesis genes in Challenger Deep samples. **a** Percentage of bacteria with DsyB and MmtN (left), and profiles of the bacterial communities harboring them (right) in depth-profiled samples determined by metagenomic analysis. Sample names are defined by size fraction and sampling depth, e.g., FL10500 is the free-living fraction at 10,500 m. FL: free-living; PA: particle-associated. **b** Absolute abundance of 16S rRNA and *dsyB* gene copies in the seawater and sediment at various depths, determined by qPCR. **c** Transcript abundance of *dsyB* in FL water and sediment samples, and of *mmtN* in sediment from different depths. **d** The effects of hydrostatic pressure on DMSP production. Left Y axis indicate strains ZYF240 (*Pseudooceanicola nanhaiensis* isolated from 8000 m seawater of the Mariana Trench), ZYF612 (*Labrenzia aggregata* isolated from 9600 m seawater of the Mariana Trench), and *Marinibacterium* sp. strain La6. Right Y axis indicates *Pelagibaca bermudensis* strain J526. **e** The survival of DMSP-producing bacteria J526 and La6 (wild type), *dsyB*^*−*^ mutant variants, *dsyB*^−^ mutants containing cloned *dsyB*, and *dsyB*^−^ mutant isolates supplied with DMSP, after incubation at 60 MPa for 10 days. Data in **e**, **f** are presented as means ± SD.

**Fig. 3:**
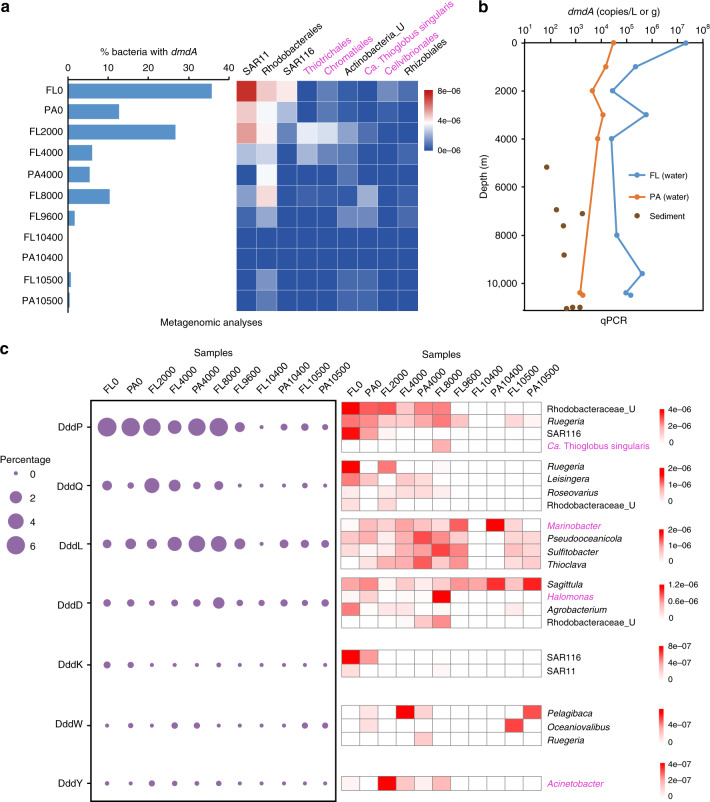
The abundance of DMSP catabolic genes in Challenger Deep water samples. **a** The relative abundance of bacterial cells containing the DMSP demethylation gene *dmdA* at different depths (left), with the top nine affiliated orders containing them shown in the heat map (right). Genera belonging to *Gammaproteobacteria* are labeled in pink; those in black font are *Alphaproteobacteria* or *Actinobacteria*. **b** Absolute abundance of *dmdA* in the seawater and sediment at various depths, as determined by qPCR. **c** The relative abundance of bacterial cells containing DMSP cleavage genes at different depths (left) with the top four affiliated genera of each gene shown in the heat map (right). Genera belonging to *Gammaproteobacteria* are labeled in pink; those in black font are *Alphaproteobacteria*. FL: free-living; PA: particle-associated.

Bacteria with DsyB or MmtN (and thus the potential to produce DMSP) were always or mostly present, respectively, in all seawater and sediment samples (Fig. 2 and Table 1), and their environmental DsyB and MmtN sequences clustered with ratified enzymes (Supplementary Figs. 4 and 5). Similar proportions of free-living (FL; 0.22–3 μm) and particle-associated (PA; >3 μm) bacteria, which dominated the metagenomes of both these fractions, contained DMSP biosynthesis and catabolic genes (Figs. 2a and 3a, and Table 1), indicating that size fractionation is not a foolproof method of separating DMSP-producing bacteria from phytoplankton. Bacteria with *dysB* were shown by qPCR and metagenomics to be relatively abundant in the surface waters (*dsyB* total abundance of 2.61 × 10^5^ copies L^−1^; 0.78–0.98% of surface water bacteria) representing ~3.49–4.38 × 10^3^ bacteria ml^−1^ of surface seawater. These numbers are comparable to those predicted from the ocean microbial reference gene catalog metagenomic database (OM-RGC)^37^ in Williams et al.^10^, at ~4.8–9.6 × 10^3^ bacteria ml^−1^. The abundance of these potential DMSP-producing bacteria initially decreased in 1000–2000 m deep seawater samples (3.46 × 10^4^ copies L^−1^; ~0.43% bacteria at these depths), but then steadily increased with depth to reach maximal levels at 10,500 m (3.95 × 10^6^ copies L^−1^; 4.03% of bacteria at 10,500 m), which were up to 15-fold higher than in the surface water (Fig. 2b, Table 1, and Supplementary Table 2). All detected *dsyB* sequences, including 37/162 metagenome assembled genomes (MAGs), were Alphaproteobacterial, mainly *Rhodobacterales*, *Rhizobiales*, and *Rhodospirillales* (Supplementary Data 1). At the genus level, *Pseudooceanicola* and *Roseovarius* were the most abundant potential DMSP producers at all depths, with much higher abundances (*P* < 0.05) in deeper waters (≥4000 m) compared to upper waters (Fig. 2a), suggesting they might be important deep water DMSP producers.

Bacteria with *mmtN* were always less abundant than those with *dsyB* in seawater metagenomes, as was also the case in coastal seawater and sediment samples in Williams et al.^10^, and their abundance did not obviously increase with seawater depth. However, the highest observed levels of bacteria with *mmtN* (~1.22% of bacteria) were found in 8000 m deep samples (Table 1). As with *dsyB*, the majority of *mmtN* homologs detected were also *Alphaproteobacteria*, belonging to bacterial genera known to produce DMSP: *Thalassospira*, *Roseovarius*, *Labrenzia*, and *Novosphingobium* (Fig. 2a; Williams et al.^10^). Furthermore, of the nine MAGs containing *mmtN*, eight were from *Alphaproteobacteria* and only one was likely from *Gammaproteobacteria* (Supplementary Data 1). Overall, the proportion of bacteria with the genetic potential to produce DMSP (containing *dsyB* and/or *mmtN*) was far higher (*P* < 0.01) in deeper waters (≥4000 m; ~2.58–5.25%) than in surface waters (~0.90–1.18%) (Fig. 2a and Table 1). Considering that the flow cytometry data of heterotrophic bacterial abundance was ~4.47 × 10^5^ and 1.86–7.56 × 10^4^ cells ml^−1^ in the surface and deep water (4000–8000 m), respectively, this equates to similar numbers of these DMSP-producing bacteria per ml seawater at the surface and below 4000 m (up to 5.27 × 10^3^ and 3.99 × 10^3^ bacteria ml^−^^1^ seawater, respectively).

Importantly, the dominant DMSP synthesis gene *dsyB* was shown to be transcribed in all tested seawater samples, and at the highest levels in the deeper waters (Fig. 2c), supporting the hypothesis that bacteria are important DMSP producers in the aphotic waters. Critically, these predictions from metagenomic and qPCR analyses are most likely an underestimation of bacterial DMSP-production potential, as several DMSP-producing bacteria, including many isolated from Challenger Deep samples, e.g., *Marinobacter* and *Erythrobacter* (accounting for ~0.02–2.6% and 0.02–2.8% at ≥4000 m, respectively), lack both *dsyB* and *mmtN* in their available genomes, and potentially contain novel DMSP synthesis genes and/or pathways (Supplementary Table 3; Williams et al.^10^).

### DMSP synthesis in deep sediment

DMSP-producing bacteria with *dsyB* and/or *mmtN* were present in all deep trench sediment samples with the highest DMSP concentrations. Furthermore, there were no plastid sequences in 16S rRNA gene amplicon sequencing data from these sediments, implying bacteria as the major producers in these environments. *dsyB* abundances ranged from ~1 × 10^3^ copies g^−1^ (in two samples) to much higher copy numbers of 0.42–1.08 × 10^5^ copies g^−1^ in the other six sediment samples (Fig. 2b and Supplementary Table 2). Compared to *dsyB*, *mmtN* abundance was lower in sediments, with the highest value of 4.07 × 10^2^ copies g^−1^ found at 6980 m (Supplementary Table 2), but this gene was still detected in most sediment samples, unlike those for the water column. Lower proportions of bacteria (~0.02–0.42%) were predicted to contain *dsyB* and/or *mmtN* in trench sediments compared to the corresponding waters (>3.20%) when qPCR was used to estimate bacterial DMSP synthesis potential (Supplementary Table 2). However, qPCR normalization to the 16S rRNA gene is not as accurate as metagenomics methods due to the existence of multiple 16S rRNA gene copies in many bacteria^38^, and the deep ocean surface sediments likely harbor far more bacteria per equivalent mass than the seawater (Fig. 2b). Indeed, *dsyB* and *mmtN* transcript abundances were far higher in all sediments than in water samples (Fig. 2c). These data indicate that DMSP-producing bacteria contribute to photic and aphotic DMSP-standing stocks, and that the significance of their contribution increases in the aphotic waters and sediments, where DMSP-producing phototrophs are far less abundant.

To identify *dsyB*- and *mmtN*-containing bacteria in the sediment, clone libraries generated from community DNA were sequenced. *mmtN* clones were all similar to those genera present in the seawater, mainly *Roseovarius* and *Labrenzia* sp. Sediment *dsyB* clones were classified into six operational taxonomic units (OTUs) at a similarity of 97%, all of which encode for proteins that cluster with functional DsyB sequences (Supplementary Fig. 6). OTU01 (57.14%) and OTU02 (19.50%) were dominant in all sediments and were homologous to *Pseudooceanicola atlanticus* and *Salipiger profundus* DsyB, respectively. OTU03, OTU04, OTU05, and OTU06 were found exclusively below 8638 m and were homologous to DsyB in *Defluviimonas* sp., *Labrenzia aggregata*, *Roseivivax pacificus*, and *Pseudooceanicola nanhaiensis*, respectively (Supplementary Fig. 6). However, the relative abundance of these potential DMSP producers, such as *Pseudooceanicola*, *Salipiger*, and *Defluviimonas*, was very low (<0.002%) in the bacterial sediment communities based on 16S rRNA gene amplicon sequencing. In contrast, the *Gammaproteobacteria Marinobacter* and *Alcanivorax* were the dominant bacteria in all sediment samples (up to ~81.60% and 48.10%, respectively) (Supplementary Fig. 7). Some *Marinobacter* isolates from this study and Williams et al.^10^ produce DMSP but lack *dsyB* and *mmtN*. This was also the case for *Erythrobacter*, which constitutes 0.06–18.30% of total bacteria in the tested sediments from 5525 m to 10,911 m (Supplementary Fig. 7). These results suggest that uncharacterized bacterial DMSP production genes and/or pathways exist and are important in these deep ocean sediments. Without knowing functional reporter genes for DMSP production in these bacteria, we are likely vastly underestimating bacterial DMSP production potential in all seawater and sediment samples.

### DMSP producing isolates in seawater

Bacterial isolation experiments were performed on all water samples (0–10,400 m) and 22 of 210 isolates produced DMSP under laboratory conditions (Supplementary Table 3). As expected^6,10^, most of these were *Alphaproteobacteria* and contained *dsyB*. However, several were *Gammaproteobacteria* and *Actinobacteria*, none of which gave *dsyB* or *mmtN* PCR products when tested with their respective degenerate primers^10^ (Supplementary Table 3), meaning they may contain novel DMSP synthesis pathways and/or genes. Seven tested DMSP-producing hadal isolates were all shown to produce DMSP when grown under physiologically relevant hydrostatic and temperature conditions (4 °C and 60 MPa) with no added methylated sulfur compounds (Supplementary Table 4). These included *Pseudooceanicola*, *Roseovarius*, *Labrenzia*, and *Erythrobacter* isolates, which represented 0.89–3.39% of hadal seawater communities (Supplementary Fig. 8), further supporting these bacteria as significant contributors to the DMSP stocks detected throughout the aphotic water column.

### Vertical distribution of DMSP catabolism genes

Given that DMSP and DMS were detected throughout the Challenger Deep water column and DMSP was concentrated in the sediment, microbial samples were analyzed for their potential to catabolize DMSP. The surface water samples harbored huge (~44.43%) bacterial populations containing the genetic potential to catabolize DMSP, equivalent to ~1.98 × 10^5^ bacteria ml^−1^ seawater. Consistent with previous studies^39–41^, *dmdA* was the most abundant DMSP catabolic gene detected in all water samples (Table 1). Bacteria with the potential to demethylate DMSP (mainly SAR11, with *Rhodobacterales* and the SAR116 clade bacteria also detected) were most abundant in the surface waters (Fig. 3a and Supplementary Fig. 9). Surface water samples contained the highest detected levels of *dmdA* (~2.22 × 10^7^ copies L^−1^), with ~35.73% and 12.73% of FL and PA bacteria, respectively, predicted to contain this gene (Fig. 3b and Table 1). In these surface FL samples, *dmdA* was ~4-fold higher than the sum total of DMSP cleavage genes (Table 1), suggesting DMSP demethylation is likely the dominant process in the surface waters.

The algal DMSP lyase *Alma1*^42^ was not detected in any trench samples, suggesting that *Alma1*-containing phototrophs are not major producers of DMS via this pathway in the tested photic and aphotic samples. In contrast, at least three or more of the seven bacterial DMSP lyase genes (*ddd*)^2^ were detected in every water sample (Fig. 3c and Table 1). *dddP* was the most abundant DMSP lyase gene in the surface waters, with 3.51 × 10^5^ copies L^−1^ detected by qPCR (Supplementary Table 2) and ~6.48% of surface ocean bacteria (~2.90 × 10^4^ bacteria ml^−1^) predicted to contain this gene—the only DMSP lyase in >1% of bacteria at the surface. The *dddK*, *dddW*, and *dddY* genes were only predicted to be in 0–0.26% of the seawater bacteria (Fig. 3c and Table 1). These metagenome values are lower than predicted from the OM-RGC database, comprised largely of surface ocean bacteria^7^. The reasons for these discrepancies are likely site- and/or season-dependent.

Given only the surface waters influence the atmosphere, then *dddP*-containing bacteria are likely key contributors to the highest detected DMS levels at these sites (Fig. 1c), a fraction of which is transferred to the atmosphere. Seawater DddP homologs clustered into four major groups (Supplementary Fig. 10). Group I was the most abundant and closely aligned to DddP from *Rhodobacteraceae* and some *Phyllobacteriaceae* bacteria. Group II proteins closely resembled *Alphaproteobacterial* DddP, with SAR116 clade being the dominant form. Groups I and II were most abundant in the surface waters. Groups III and IV had multiple representatives, including *Alphaproteobacteria*, *Gammaproteobacteria*, *Betaproteobacteria*, and *Actinobacteria* (Supplementary Fig. 10), suggesting lateral gene transfer event/s^43^. *dddP* was found in 43% of MAGs (69), predicted to be *Alphaproteobacteria*, *Gammaproteobacteria*, *Acidimicrobiia*, *Bacteroidia*, SAR324, *Nitrososphaeria*, and *Anaerolineae* (Supplementary Data 1).

Bacterial DMSP catabolism was also likely important in aphotic 2000–8000 m deep waters, with 14.28–36.88% of bacteria predicted to contain a DMSP catabolic gene. *dmdA* was still the dominant gene at these depths, predicted to be present in 5.43–26.66% of bacteria, but its relative abundance decreased with depth (Fig. 3a, b, Table 1, and Supplementary Table 2). Howard et al.^40^ detected no *dmdA* genes in 500–4000 m deep Pacific Station Aloha seawater samples, likely due to lower sequencing depth (8.86–11.18 Mb)^44^ compared to sequencing performed here (13.67–16.54 Gb) and/or the more extensive *dmdA* gene probe sequences used in this study (Supplementary Table 5). The abundance of *Alphaproteobacterial dmdA* generally decreased with water depth, whereas those *dmdA* sequences from *Gammaproteobacteria* and *Actinobacteria* did not vary with depth (Fig. 3a). Of 162 MAGs, 58 contained *dmdA*, likely from *Alphaproteobacteria*, *Gammaproteobacteria*, *Acidimicrobiia*, SAR324, and *Nitrososphaeria* (Supplementary Data 1). Interestingly, the relative abundance of bacteria with DMSP lyases significantly increased in these deeper waters (2000–8000 m), with cumulatively more *ddd* genes observed in metagenomes from 4000 m to the trench bottom, compared to *dmdA* (Table 1). DddP was still the dominant DMSP lyase in the 2000–8000 m deep waters (averaging 4.84%), but DddQ (up to 3.55%), DddL (up to 4.61%), and DddD (up to 1.61%) were better represented in these waters compared to the surface waters (Fig. 3c and Table 1). Seawater DddQ sequences were most similar to those in the *Rhodobacteraceae*, including *Ruegeria*, *Leisingera*, and *Roseovarius* (Fig. 3c). All DddL sequences were homologous to *Gammaproteobacteria*, represented by *Marinobacter*. In comparison, the DddD homologs varied through the water column, with surface waters containing *Alphaproteobacterial Sagittula* homologs, and *Gammaproteobacterial Halomonas* homologs being dominant in 8000 m samples (Fig. 3c). *dddQ* (8 MAGs), *dddL* (37 MAGs), *dddD* (47 MAGs), *dddK* (2 MAGs), and *dddW* (7 MAGs) were also represented in the 162 MAGs (Supplementary Data 1). Although the relative abundance and therefore the likely importance of DMSP lyase genes in these microbes increased in the 2000–8000 m deep waters compared to the surface, their absolute abundance did not necessarily increase, due to ~4-fold more bacteria being present in the surface waters, e.g., *dddP* copies L^−1^ were most abundant in the surface waters based on qPCR results (Supplementary Table 2). Furthermore, 43 of 210 bacterial isolates had the ability to cleave DMSP, 29 being from the 2000–8000 m deep water samples (Supplementary Table 6).

Metagenomics showed there to be a steep decline in DMSP catabolic potential in 9600 m deep waters and below, with no *ddd* or *dmdA* gene predicted in more than 1.65% of the bacteria. Indeed, *dmdA* was absent in 10,400 m deep metagenomes. Despite this, qPCR data showed no correlation between *dmdA* and *dddP* absolute gene abundance and depth, other than the highest levels being in the surface waters. It is possible that there are more bacteria in the deepest waters that were not assayed by flow cytometry. Perhaps in these waters there is a stronger requirement to synthesize and store DMSP for its anti-stress properties than to catabolize it; thus, a lower proportion of bacteria in the community would have this ability.

In contrast to most seawater samples, in which *dddP* and *dmdA* were abundant, these DMSP catabolic genes were at their lowest detected levels in the deep ocean sediments that contained the highest DMSP concentrations. The DMSP lyase *dddP* was undetected by qPCR in all sediment samples, and only 8.30 × 10^1^–2.18 × 10^3^ *dmdA* copies g^−1^ were observed in the hadal sediments (Fig. 3b and Supplementary Table 2). This could suggest that the primers are not detecting deep sediment variants of these genes, that they are scarcely present in these environments and are thus not as important in hadal sediments as they are in seawater, or that other Ddd enzymes and/or isoform enzymes exist in bacteria in these sediments. Further work measuring DMSP synthesis and catabolic process rates and/or transcript/protein abundance is required to better establish the importance of bacteria in these processes throughout the Challenger Deep water and sediment samples, and to test the hypotheses raised here.

### A role for DMSP in bacterial hydrostatic pressure tolerance

As *dysB* abundance and transcripts increased with depth and DMSP catabolic potential was less prominent in the deepest seawater and sediment communities, the hypothesis that DMSP may help organisms to survive under deep ocean hydrostatic stress was tested. DMSP-producing bacteria, isolated from 8000 m (*P. nanhaiensis* ZYF240) and 9600 m deep (*L. aggregata* ZYF612) Mariana Trench seawater, both exhibited significantly enhanced DMSP production per colony forming unit (CFU) with increasing pressure (Fig. 2d) with no added methylated sulfur compounds. Another two isolates (*Pelagibaca bermudensis* J526 and *Marinibacterium* sp. La6) from surface seawater also showed the same result. Furthermore, DMSP-producing bacteria (wild type) could survive deep ocean pressure (60 MPa) far better than *dsyB*^−^ mutant strains unable to produce DMSP, with the phenotype being restored by cloned *dsyB* or when DMSP was provided (Fig. 2e). This provides convincing evidence in at least some marine bacteria for a new role for DMSP in protecting cells against the high hydrostatic pressures that exist in the deep ocean.

## Discussion

**Fig. 4:**
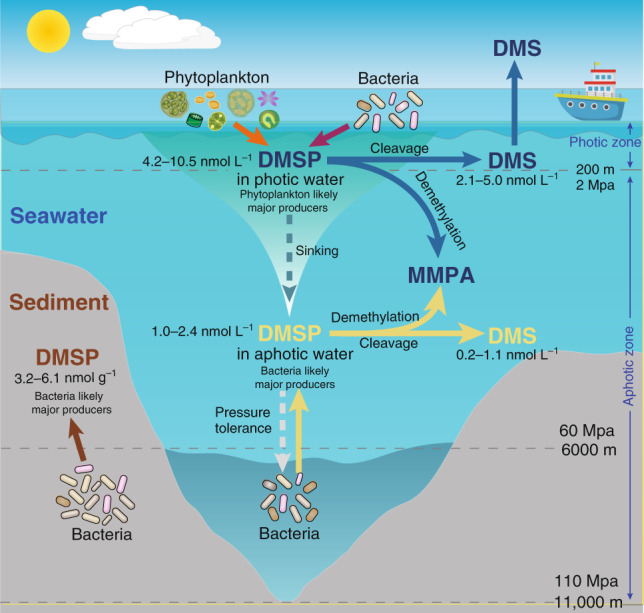
The proposed cycling of DMSP throughout the water column. Phytoplankton are the major contributors to DMSP production in the photic zone, whereas in aphotic zones, where no sunlight penetrates, heterotrophic bacteria likely contribute significantly to DMSP production. DMSP produced in the surface waters can sink to lower levels. Sedimentary DMSP levels are two to three orders of magnitude higher, per equivalent mass, than the seawater, and are also most likely produced by bacteria. The relative abundance of DMSP catabolic genes was lowest in the deepest water and sediment samples, and DMSP can play a role in protecting bacteria against increased hydrostatic pressure in such deep waters and sediment. DMSP and DMS produced in the surface water is labeled in blue. Deep-ocean DMSP and DMS is in yellow and sedimentary DMSP is labeled in brown. Values in this figure represent DMSP or DMS concentration ranges determined by this study.

Until recently, only photosynthetic eukaryotes were thought to produce DMSP^2,6^, thus most DMSP research has focused on euphotic zones. The discovery of DMSP biosynthesis in marine bacteria and genes for its synthesis and catabolism in prokaryotes and eukaryotes^2,10^ made it possible to evaluate the role of these microorganisms in DMSP production and cycling in unexplored environments, e.g., through the depth profile of Earth’s deepest ocean site: the Challenger Deep. Phytoplankton likely dominate DMSP production in the photic zone, but heterotrophic DMSP-producing bacteria should be considered significant contributors given their abundance and *dsyB* transcript levels. In deeper aphotic environments (both water and sediment) with negligible phytoplankton, bacteria likely have a more substantial contribution to DMSP production (Fig. 4). Considering that aphotic waters and sediment represent far larger mass than the photic zone, and that DMSP is consistently detected at ~1.6 × 10^−3^ nmol ml^−1^ levels throughout the aphotic waters and is also hugely concentrated in the sediment, then the global DMSP stock of the deep ocean is potentially more considerable than in the photic waters, which have the highest seawater concentrations. This DMSP, whether bacterial- or particle-based, supports enormous populations of heterotrophs via DMSP demethylation and lysis pathways, particularly in the water column. However, the relative importance of known DMSP synthesis pathways to microbial communities is highest, whereas that of DMSP catabolic pathways is lowest in the deepest seawater and sediment samples. Even so, this does not appear to result in higher DMSP levels in the deep seawater samples, possibly due to low growth rates or to unexplored DMS and DMSP transformations, e.g., the formation of the metabolite dimethylsulfoxonium propionate^45^ or the existence of further novel bacterial DMSP lyases^46^ in the deep ocean. Moreover, much of the DMSP produced by bacteria in the deep ocean may be stored and used as an anti-stress molecule in these environments. Indeed, our data are consistent with some marine bacteria producing DMSP to protect against the hydrostatic pressure of the deep ocean environment. Ultimately, this study challenges the beliefs that DMSP and DMS production are mainly photic processes and suggests that the aphotic production of these influential sulfur compounds, particularly by bacteria, is globally significant. It is important that future studies of deep ocean DMSP and DMS cycling consider the production and turnover rates of these compounds to enable a better understanding of the fluxes at play and, ultimately, their global significance.

## Methods

### Sample collection

Seawater (~200 L) at 0, 2000, 4000, and 8000 m was collected from the Challenger Deep of the Mariana Trench (11°21.847’N, 142°20.775’E) aboard the R/V *Dong Fang Hong 2* in March 2017. Seawater was filtered serially through 3 μm (TSTP, 142 mm, Millipore) and 0.22 μm (GTTP, 142 mm, Millipore) polycarbonate membranes. All filters were stored in liquid nitrogen on board, and at −80 °C in the laboratory. The communities collected on the 3 and 0.22 μm filters were designated as PA and FL fractions, respectively. Metagenome samples at 0, 4000, 9600, 10,400, and 10,500 m were treated in the same way as our previous study^47^. Values calculated for gene abundance at 0 and 4000 m were the mean of two replicates from different cruises but the same station (September 2016 and March 2017). Replicates from 2000 and 8000 m were taken on the same cruise (March 2017). The deep surface sediment samples (~10,910 m) were collected from the Mariana Trench (11°19.5132’N, 142°11.2906’E) during research cruise TS-03 in March 2017, using the lander “TianYaHao”. Sediments at 5525, 6980, 7110, 7670, and 8638 m were collected from the Mariana Trench using a box corer in July 2016. Detailed location information is shown in Fig. 1a and Supplementary Table 1.

### Picoplankton abundance analysis

Water samples used for picoplankton abundance analysis were collected at depths of 4, 100, 200, 454, 868, 2286, 4000, 6264, and 8267 m from the Challenger Deep of the Mariana Trench (11°20.285’N, 142°23.964’E) aboard the R/V *Dong Fang Hong 2* in November 2017. Water samples (2 ml) were immediately fixed with paraformaldehyde (final concentration 1%, v/v) for 1 h in the dark at room temperature and then stored in liquid nitrogen on board. The abundances of *Synechococcus*, *Prochlorococcus*, picoeukaryotes, and heterotrophic bacteria were measured with a BD FACSJazz flow cytometer (Becton-Dickinson, USA)^48^ in the laboratory.

### DNA and RNA preparation

DNA was extracted from water samples as previously described^47^ using phenol–chloroform extraction method. Sedimental DNA at depths of 5525–10,911 m was isolated following the DNeasy PowerSoil Kit (Qiagen). RNA was extracted using the Trizol reagent (Sigma) and cleaned using the Direct-zol RNA MiniPrep kit (Zymo Research). DNA was removed using the TURBO DNA-free kit (Thermo Fisher Scientific). Successful DNA removal was confirmed by 16S rRNA gene PCR.

### 16S rRNA gene amplicon sequencing and metagenomic sequencing

Water and sediment DNA was sent to Majorbio Bio-pharm Technology Co., Ltd (Shanghai, China) for 16S rRNA gene amplicon sequencing. Primers 515F and 806R (Supplementary Table 7) were used to amplify the V4 region of the 16S rRNA gene. Sequencing was performed on the Illumina Miseq PE300 platform (MiSeq Reagent Kit v3). Water DNA samples at 0, 2000, 4000, and 8000 m, except for the PA fraction of 2000 and 8000 m due to an insufficient quantity of DNA, were sent to Novogene Bioinformatics Technology Co., Ltd (Beijing, China) for metagenomic sequencing. Libraries were prepared without any amplification step for each sample. Metagenomic shotgun sequencing was performed on the Illumina HiSeq X-Ten platform, with 2 × 150 bp paired-end reads.

### Quantitative PCR and reverse transcription qPCR

The abundance of bacterial 16S rRNA genes was quantified using qPCR, with primers Eub338F and Eub518R (Supplementary Table 7). Primers used for quantifying genes involved in DMSP synthesis and degradation are listed in Supplementary Table 7. The construction of qPCR standards and the qPCR assay was performed as described in a previous study^46^. For reverse-transcription qPCR, 9 µl of purified RNA was mixed with 1 µl random hexamer primers (Invitrogen). The reaction system was incubated at 70 °C for 5 min and cooled on ice, before mixing with 1 µl dNTPs (10 mM), 4 µl of M-MLV RT 5× buffer (Promega), 0.8 µl of M-MLV reverse transcriptase (Promega), 0.4 µl of RNase inhibitor (40 U µl^−1^, Roche), and 3.8 µl of Diethyl Pyrocarbonate (DEPC) water. This was then incubated at 42 °C for 1 h and the obtained cDNA was stored at −80 °C.

### Construction and analysis of *dsyB* and *mmtN* clone libraries

To study the diversity of *dsyB* and *mmtN* in sediment, clone library sequencing was performed using their respective degenerate primers (Supplementary Table 7) on sediment from 5525, 6980, 8638, 10,908, and 10,909 m deep. This was instead of the metagenomic sequencing performed on water samples, as there was not enough material. The clone library construction method followed a procedure similar to that described in Yin et al.^49^. Briefly, the PCR amplicon products were inserted in the pUCm-T vector and transformed into *Escherichia* *coli* JM109 competent cells (purchased from Beijing Biomed Genetic Technology Co., Ltd). Positive clones were picked and sequenced. The *dsyB* OTUs were determined with a nucleotide similarity of 80% by Mothur. Representative sequences of each OTU were translated into protein sequences and were used for phylogenetic tree construction.

### Taxonomic analyses of sequencing data

The 16S rRNA gene sequences were processed with the pipeline of UPARSE^50^. OTUs were clustered at a 97% similarity level. For metagenome sequencing, filtered reads assembly, gene prediction and annotation, and gene abundance calculations were performed as described in our previous study^47^.

### Searching for DMSP cycling genes in metagenome data

To identify DMSP production and degradation genes, alignments of ratified sequences of all genes of interest (listed in Supplementary Table 5) were used to build HiddenMarkov Model (HMM) profiles to perform HMM searches as described in Curson et al.^7^. Predicted DMSP cycling proteins with an *E*-value of ≤10^−50^ were kept and placed into phylogenetic trees, to determine other potential functional homologs. Phylogenetic trees were constructed using MEGA version 7.0 and included non-functional sequences used as outgroups. Sequences that clustered with non-functional sequences were removed and the rest were further analyzed by BLASTp searches against the RefSeq database at NCBI, followed by manual annotation to verify that the top hits were the target protein. To account for variation in bacterial cell numbers between samples, the percentage of bacteria harboring *dsyB* was normalized to the total microbial community using the single-copy housekeeping gene *recA*, assuming one copy of *dsyB* per cell. The HMM profile for RecA was downloaded from FunGene (http://fungene.cme.msu.edu/) and sequences with an *E*-value of ≤10^−50^ were retained. The percentage of cells containing a particular gene of interest was calculated as (gene homologs × 100)/*recA*^40^. The annotation of DMSP-related sequences was performed using BLASTp with an *E*-value cutoff of 10^−5^ against NCBI-nr databases for taxonomic analysis and only the best hits were retained. Taxonomy assignment was performed in MEGAN software^51^ based on the BLAST results of the nr database using the lowest common ancestor algorithm.

### MAG construction

Reads of different water depths were analyzed for metagenomic binning separately with the metaWRAP-Assembly module using MegaHit (version 1.1.2)^52^. Metagenomics binning software MaxBin2 (version 2.2.4)^53^, metaBAT2 (version 2.12.1)^54^, and CONCOCT (version 0.4.0)^55^ were used to produce three MAGs. These were consolidated into a single, stronger MAG, which was further improved with reassembly using Reassemble_bins. Completeness and contamination of MAGs were assessed using CheckM^56^, and MAGs with a completeness > 50% and contamination < 10% were considered. Taxonomy of each bin was determined by CheckM, further confirmed by Taxator-tk (version 1.3.3e)^57^. DMSP-related gene sequences (Supplementary Table 5) were used as query sequences to perform BLASTp against MAGs sequences. An identity value of 40% and coverage of 70% were used as thresholds to capture the functional proteins.

### DMSP concentration measurement

Seawater samples were collected from Challenger Deep (0, 50, 100, 120, 150, 200, 6050, and 8320 m) in March 2017 and (500, 1000, 1500, 2000, 3000, 4000, and 10,400 m) in September 2016. To measure DMSP content, 10 ml seawater was put into 15 ml tubes and 100 μl 50% H_2_SO_4_ was added to remove pre-existing DMS and preserve DMSP for later analysis^58^. For the September 2016 cruise, these samples were put at room temperature for at least 1 h and then stored at −20 °C while on board. For the March 2017 cruise, samples were stored at 4 °C. When samples were brought to the laboratory, those stored at −20 °C were thawed overnight at room temperature. To cleave DMSP into DMS, 2 ml seawater was put into 10 ml vials and mixed with 200 μl NaOH (10 M), and crimp sealed. Due to this non-standard procedure of sample storage, we tested whether freezing DMSP and DMS samples in seawater with H_2_SO_4_ overestimates DMSP, due to the inhibition of DMS oxidation. Three fresh seawater samples collected from Luxun Park in Qingdao (Shandong, China) were halved and H_2_SO_4_ was added to both halves. One was stored at room temperature and another was frozen at −20 °C. We found that this freezing process did not lead to an overestimation of DMSP (Supplementary Fig. 11). However, it is possible that a portion (<1 nM) of the seawater DMSP measured in this study may not be actually DMSP but some other organic sulfur compound that produces DMS upon alkaline hydrolysis^29^. For sediment samples, 0.1 g sediment was placed into 2 ml vials,and 100 μl water and 100 μl NaOH (10 M) were added before crimp sealing. Reactions were stored in the dark for 24 h. The DMS released was quantified by a purge-and-trap gas chromatography (GC) system or GC auto-injection, respectively, using a flame photometric detector (Agilent 7890B GC fitted with a 7693A autosampler) and an HP-INNOWax 30 m × 0.320 mm capillary column (Agilent Technologies J&W Scientific). All assays were carried out in triplicates.

### DMS and Chl-a concentration measurements

The concentrations of DMS in the seawater were measured immediately after collection on board, using a purge-and-trap GC system. For Chl-*a* analysis, seawater samples were filtered through a 47 mm Whatman GF/F filter immediately after collection on board. The filters were subsequently stored at −20 °C. When samples were brought to the laboratory, the filters were soaked in 90% acetone in the dark, overnight, to extract Chl-*a*^59^. After centrifugation, a F4500 fluorescence spectrophotometer (Hitachi, Japan) was used to determine the Chl-*a* concentration.

### Isolation of DMSP-producing and -degrading bacteria

A total of 210 single colonies isolated from different water depths of Challenger Deep were purified and tested for DMSP production. Bacterial isolates were cultivated in Marine Broth (MB) medium (per liter seawater: 1 g yeast extract, 5 g peptone, 0.01 g ferric phosphate pH 7.6) for 24 h. To determine DMSP content by alkaline lysis, 200 μl of culture and 100 μl of NaOH (10 M) were mixed in 2 ml vials. Vials were crimped immediately, incubated at room temperature overnight in the dark and headspace monitored by GC. Isolates possessing a DMS peak were retained to perform secondary screening. These isolates were cultivated in marine basal medium (MBM) minimal medium (salinity 35 PSU)^60^ supplemented with a mixed carbon source (10 mM from a 1 M stock of 200 mM succinate, glucose, pyruvate, sucrose, and glycerol). Hereafter, this medium with the above ingredients was designated as MBM medium. We cultivated bacteria in triplicate under two conditions (i.e., adding 0.5 mM l-Met supplied with 10 mM NH_4_Cl as a nitrogen source, and no l-Met added with lower nitrogen levels of 0.5 mM NH_4_Cl). After 24 h, 200 μl of culture (in triplicate) was added to 2 ml vials and was used to test for the presence of DMS directly by GC. In parallel, another 200 μl of culture was added to 2 ml vials and mixed with 100 μl 10 M NaOH (in triplicate). The latter vials were incubated at room temperature for overnight in the dark and the DMS was detected by GC. The emission of DMS after alkaline hydrolysis minus the DMS detected before alkaline hydrolysis was considered to be DMSP production. All 210 bacterial isolates were also screened for DMSP-dependent DMS production. For this, isolates were grown in vials containing 200 μl MBM supplemented with 1 mM DMSP for 24 h, alongside appropriate controls. The DMS in the headspace was quantified as above.

To establish whether the DMSP-producing bacteria harbored *dsyB* and/or *mmtN*, their genomic DNA was used in PCR with their degenerate primers to these DMSP synthesis genes (Supplementary Table 7). The PCR system and amplification conditions were the same as in Williams et al.^10^.

### DMSP production analysis of hadal isolates

Triplicate cultures of the isolates (100 μl, OD_600_ = 1.0) were added to 2 ml MBM medium with lowered nitrogen levels (0.5 mM NH_4_Cl) and no l-Met added. They were incubated at 4 °C, 60 MPa for 35 days. High-pressure incubations were conducted in stainless steel reactors (380 ml, maximum pressure 60 MPa; Nantong Feiyu Oil Science and Technology Exploitation, China). Pressure was delivered by water using a manual pump. To quantify DMSP, once the incubation was finished, 200 μl of culture (in triplicate) was added to 2 ml vials and was used to test for the presence of DMSP by GC as described above.

### The effects of pressure on bacterial DMSP production

*P. nanhaiensis* ZYF240 and *L. aggregata* ZYF612 were isolated from 8000 and 9600 m seawater of the Mariana Trench, respectively. Another two surface seawater strains J526 (*P. bermudensis*, purchased from DSMZ-German Collection of Microorganisms and Cell Cultures GmbH) and La6 (*Marinibacterium* sp. provided by Professor Colin Murrell of University of East Anglia) were also used in this experiment. These were incubated in MBM medium with reduced nitrogen (0.5 mM NH_4_Cl) and no added methylated sulfur compounds under different pressure conditions, i.e., 0.1, 10, 20, 30, and 40 MPa at room temperature for 4 weeks. After incubation, DMSP production was determined by GC as described above. As high pressures may reduce bacterial growth, DMSP production was normalized to CFUs. Bacterial colonies were counted by spreading serially diluted cultures on Marine Agar (MA) plates. All assays were performed in triplicate.

### Pressure effect on wild and *dsyB*^−^ mutant bacterial growth

*dsyB*^−^ mutant and complemented strains of the DMSP producers J526 (*P. bermudensis*) and La6 (*Marinibacterium* sp.) were used to investigate whether DMSP production provides protection against high pressure. The *dsyB* in-frame deletion mutant of a J526 Rif^R^-derivative strain was constructed by a double-crossover allelic exchange, using the suicide vector pK18mob*sacB*. The primers used are shown in Supplementary Table 7. To construct the Δ*dsyB* mutant of J562 Rif^R^, two primer pairs (J526-DdsyB-UO/UI and J526-DdsyB-DI/UO) were used to amplify the *dsyB* upstream and downstream fragments, respectively. Both fragments were purified and fused in a subsequent PCR reaction using primers J526-DdsyB-UO and J526-DdsyB-DO. The fused segment was cloned into the suicide vector pK18mobsacB, transformed into *E. coli* 803, and mobilized into J562 Rif^R^ by triparental mating, using the helper plasmid pRK2013, as in Curson et al.^6^. The transconjugants with a single-crossover insertion in the chromosome were obtained by screening on MA with rifampicin (20 μg/ml) and kanamycin (20 μg/ml). Allelic exchange between the chromosomal gene and the mutagenized plasmatic copy was achieved in a second crossover event, which was counter-selected on MA containing 10% (w/v) sucrose to cause the excision of the suicide vector from the chromosome. The resultant Δ*dsyB* mutant of J562 Rif^R^ was selected by kanamycin (20 μg/ml) sensitivity and was confirmed by PCR, sequencing, and by the loss of DMSP production. The Δ*dsyB* mutant of La6 Rif^R^ was constructed in the same way, using La6-specific primers (Supplementary Table 7). Genetic complementation was performed using cloned *dsyB* from *L*. *aggregata* IAM12614 in the expression vector pLMB509-GFP^61^, confirmed to be functional by Curson et al.^6^. Cloned *dsyB* was mobilized into Δ*dsyB* mutants by triparental mating with the helper plasmid pRK2013, as in Curson et al.^6^. The transconjugants were obtained on MA with rifampicin (20 μg/ml) and gentamycin (20 μg/ml), and the complemented strains of Δ*dsyB* mutants were confirmed by PCR, sequencing, and phenotype of partial recovery of DMSP production capability.

Wild-type, *dsyB*^*−*^ mutant, and complementary strains of J526 and La6 were cultured in MBM medium for 2 days. After adjusting to the same OD_600_ value, 50 μl were transferred into 450 μl fresh MBM medium with low nitrogen (0.5 mM NH_4_Cl) and no added l-Met. To investigate whether the phenotype of the *dsyB*^−^ mutant could be restored by exogenous DMSP, additional DMSP (1 mM) was added into *dsyB*^*−*^ mutant cultures. All cultures were incubated under 28 °C, 60 MPa for 10 days. Cultures (100 μl) were serially diluted ten-fold and then 10 μl dotted on MA plates. These plates were incubated at 28 °C for 4 days. The growths of wild-type, *dsyB*^*−*^ mutant, and complementary strains were observed by counting and compared. All assays were performed in triplicate.

### Reporting summary

Further information on research design is available in the Nature Research Reporting Summary linked to this article.

## Supplementary information

Supplementary Information

Description of Additional Supplementary Files

Supplementary Data 1

Reporting Summary

## Data Availability

Sequence data for metagenome of water samples from September 2016 (PRJNA412741) and March 2017 (PRJNA541485), and 16S rRNA reads of water samples (PRJNA413447) and sediment samples (PRJNA416963) have been deposited in the NCBI Sequence Read Archive.
