# Supplementary material for: Bacteria are important dimethylsulfoniopropionate producers in marine aphotic and high-pressure environments
Source: Nat Commun. 2020 Sep 16;11:4658. doi: 10.1038/s41467-020-18434-4 (PMC7494906; doi:10.1038/s41467-020-18434-4)
Supplement: Supplementary file 1 — Supplementary Information [file 41467_2020_18434_MOESM1_ESM.pdf]

Supplementary Information

Bacteria are important producers of dimethylsulfoniopropionate in aphotic waters

Zheng *et al*.

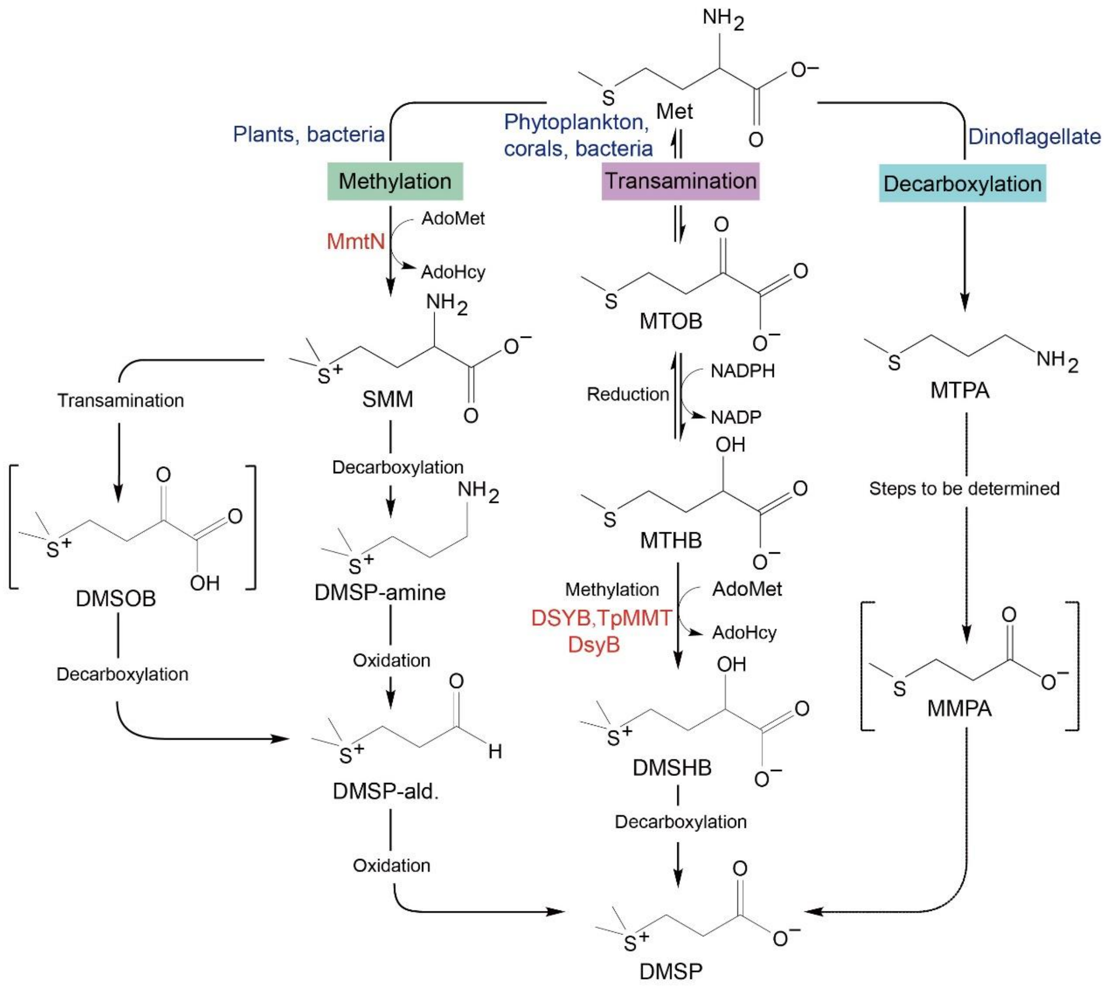

Supplementary Figure 1. The three DMSP synthesis pathways (modified from Zhang et al.[1]; Curson et al.[2]). The pathways are named after the primary modification of methionine. Known enzymes involved in the synthesis of DMSP are coloured in red. AdoMet, S-adenosylmethionine; AdoHcy, S-adenosyl-L-homocysteine; DMSP-ald., DMSP-aldehyde; MMPA, methylmercaptopropionate. MTPA, 3-methylthiopropylamine; MTOB, 4-methylthio-2-oxobutyrate; MTHB, 4-methylthio-2-hydroxobutyrate; DMSHB, 4-dimethylsulfonio-2-hydroxybutyrate; SMM, S-methyl-L-methionine; DMSP-Ald, 3-dimethylsulfoniopropionaldehyde; DMSOB, 4-dimethylsulfonio-2-oxobutyrate.

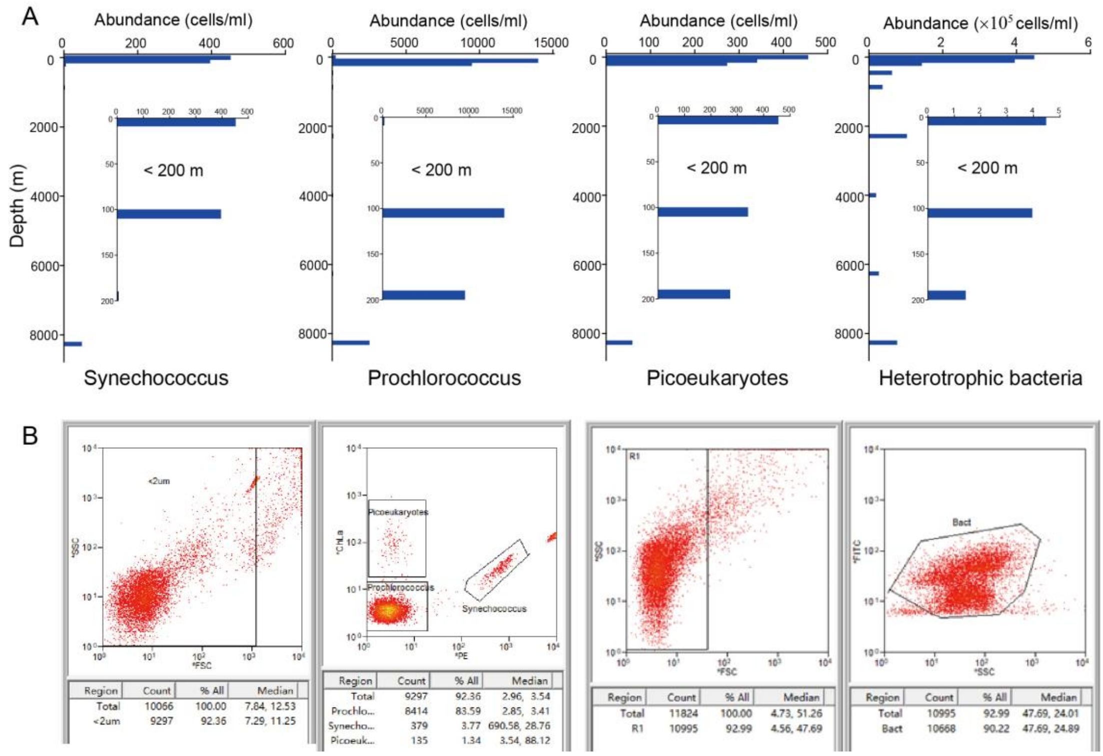

Supplementary Figure 2. Vertical distribution of picoplankton (*Synechococcus*, *Prochlorococcus*, picoeukayotes and heterotrophic bacteria) abundance (A) in the Mariana Trench and their cytogram (B) determined by flow cytometry. The abundance levels in the photic waters (above 200 m) are enlarged. The values for heterotrophic bacteria were used to estimate the numbers of DMSP-producing bacteria in samples.

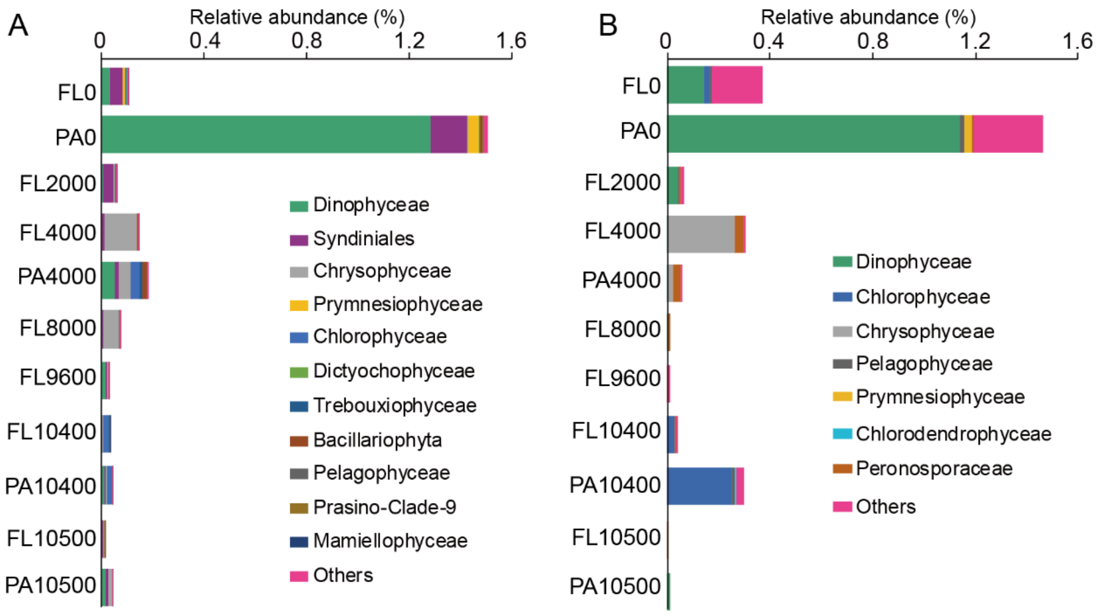

Supplementary Figure 3. Phytoplankton communities at class level throughout the water column, determined by metagenomic analysis (A) and 16S rRNA gene amplicon analysis (B). The percentage of phytoplankton in the total microbial community was calculated according to number of reads apportioned to phytoplankton dividing by total reads number. Values of 0-8,000 m were from two replicates for metagenomics (A) and three replicates for 16S rRNA gene amplicon sequencing (B).

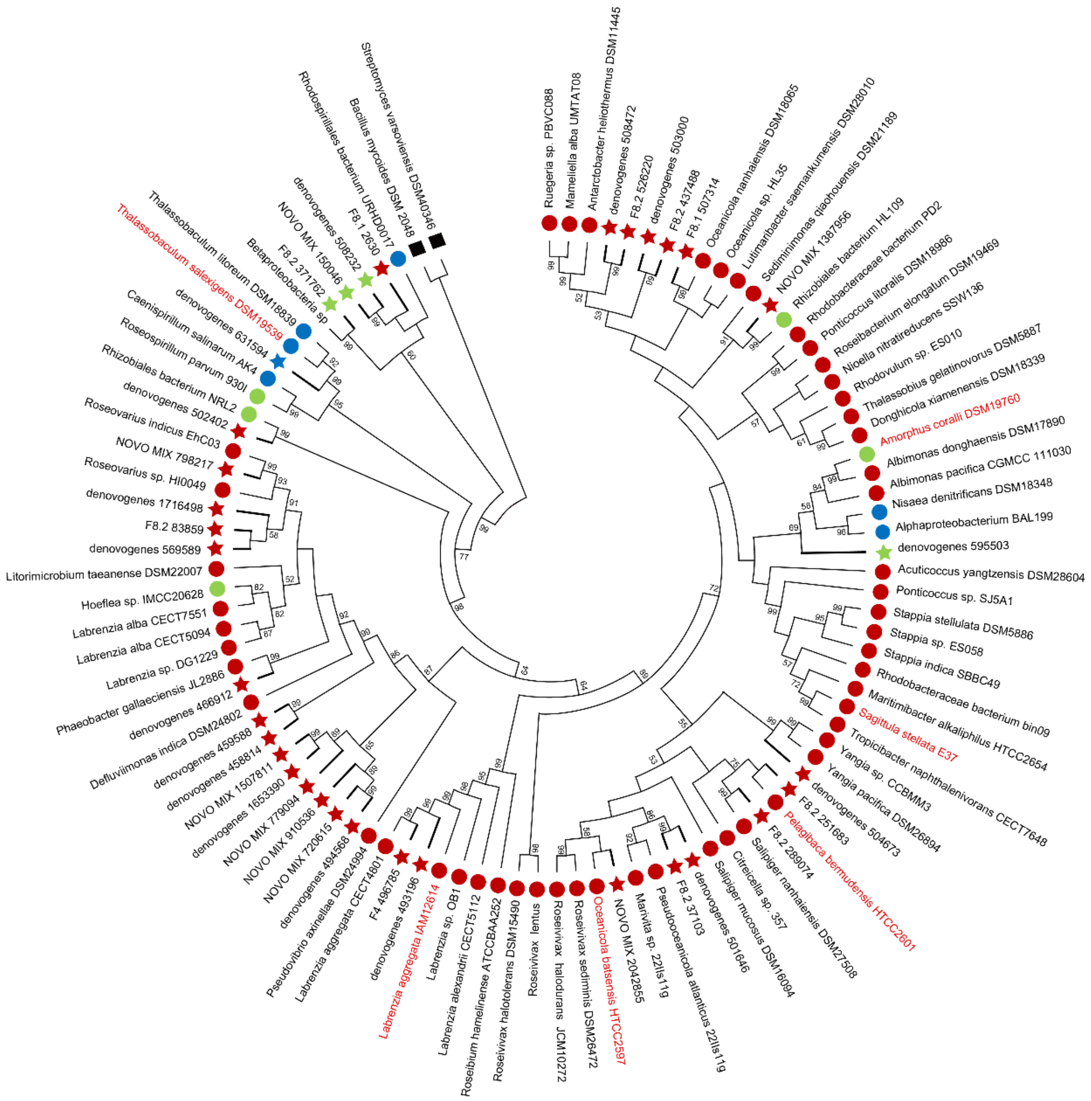

Supplementary Figure 4. Neighbour-joining phylogenetic tree of DsyB proteins predicted from metagenomic data. DsyB sequences according to Curson et al.[2] were used as reference sequences. A total of 100 sequences were used to construct this tree. DsyB sequences experimentally confirmed to produce DMSP are labeled in red. Stars indicate sequences from our metagenomic data. Circles with different colours indicate proteins from different bacterial groups. Red: *Rhodobacterales*; Green: *Rhizobiales*; Blue: *Rhodospirillales*. Squares represent non-functional DysB proteins used as the outgroup (*Bacillus mycoides* DSM 2048 and *Streptomyces varsoviensis* DSM40346).

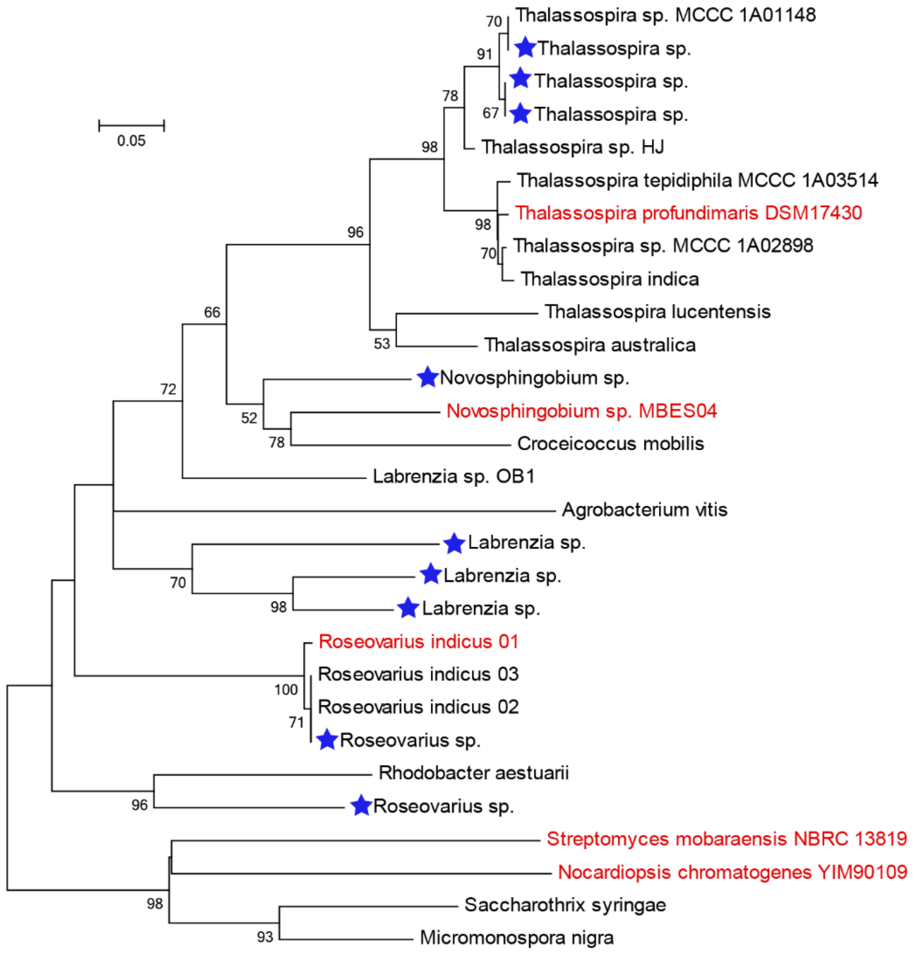

Supplementary Figure 5. Neighbour-joining phylogenetic tree of MmtN proteins, predicted from metagenomic data. MmtN sequences according to Williams et al.[3] were used as reference sequences. Blue stars represent MmtN sequences from this metagenomic data. MmtN sequences functionally ratified are labeled in red. A total of 29 sequences were used to construct this tree.

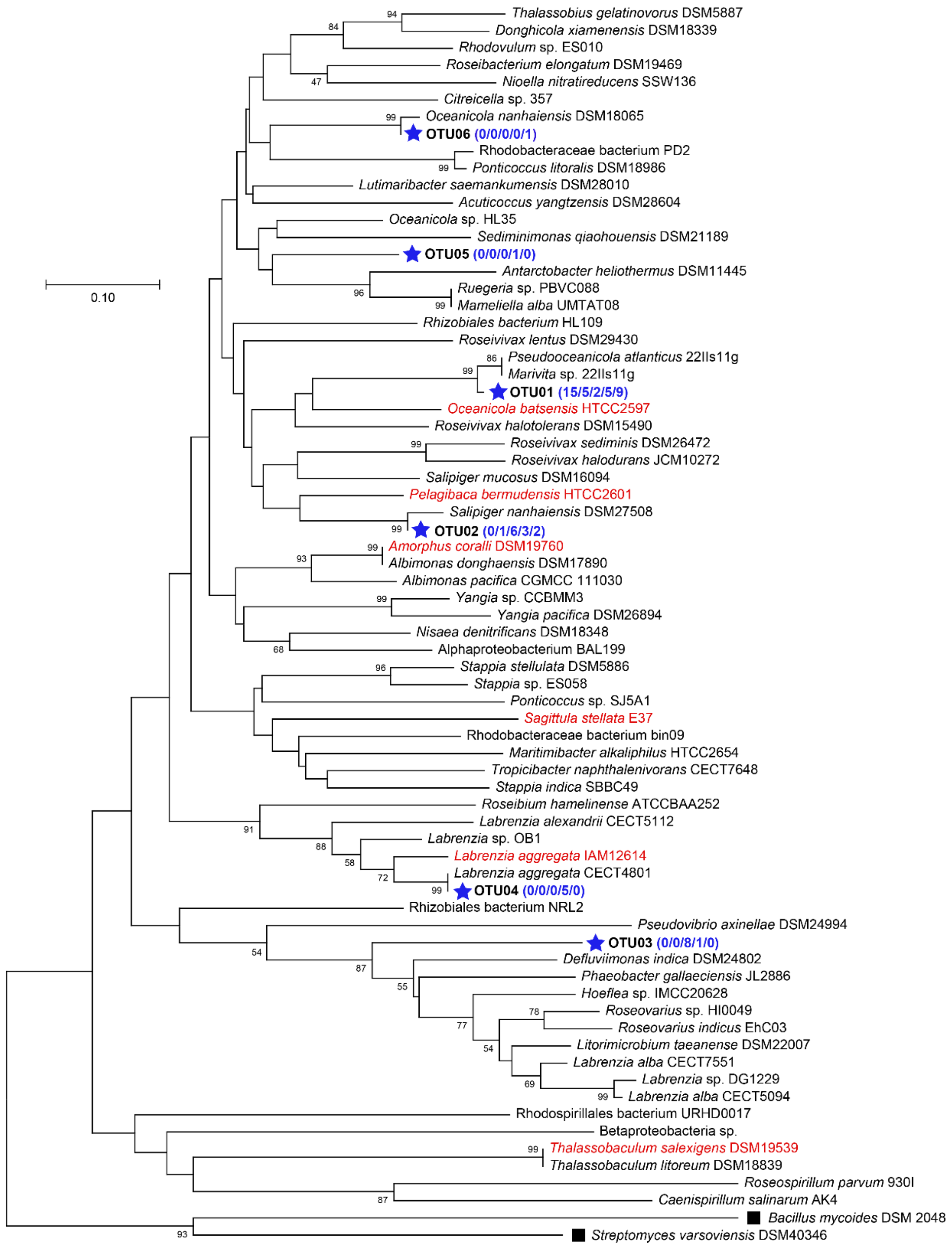

Supplementary Figure 6. Phylogenetic tree of DsyB protein OTU sequences from sediments determined by clone library sequencing. This tree was constructed from 72 sequences including experimental and sequences from the NCBI RefSeq non-redundant protein database. Stars and squares represent DsyBs from this study and non-functional DsyBs, respectively. The numbers in blue indicate the OTU number in the sediments at depths of 5,525, 6,980, 8,638, 10,908 and 10,909 m, respectively. This

neighbor-joining tree was made with the Passion model. DsyB sequences experimentally confirmed to produce DMSP were labeled in red.

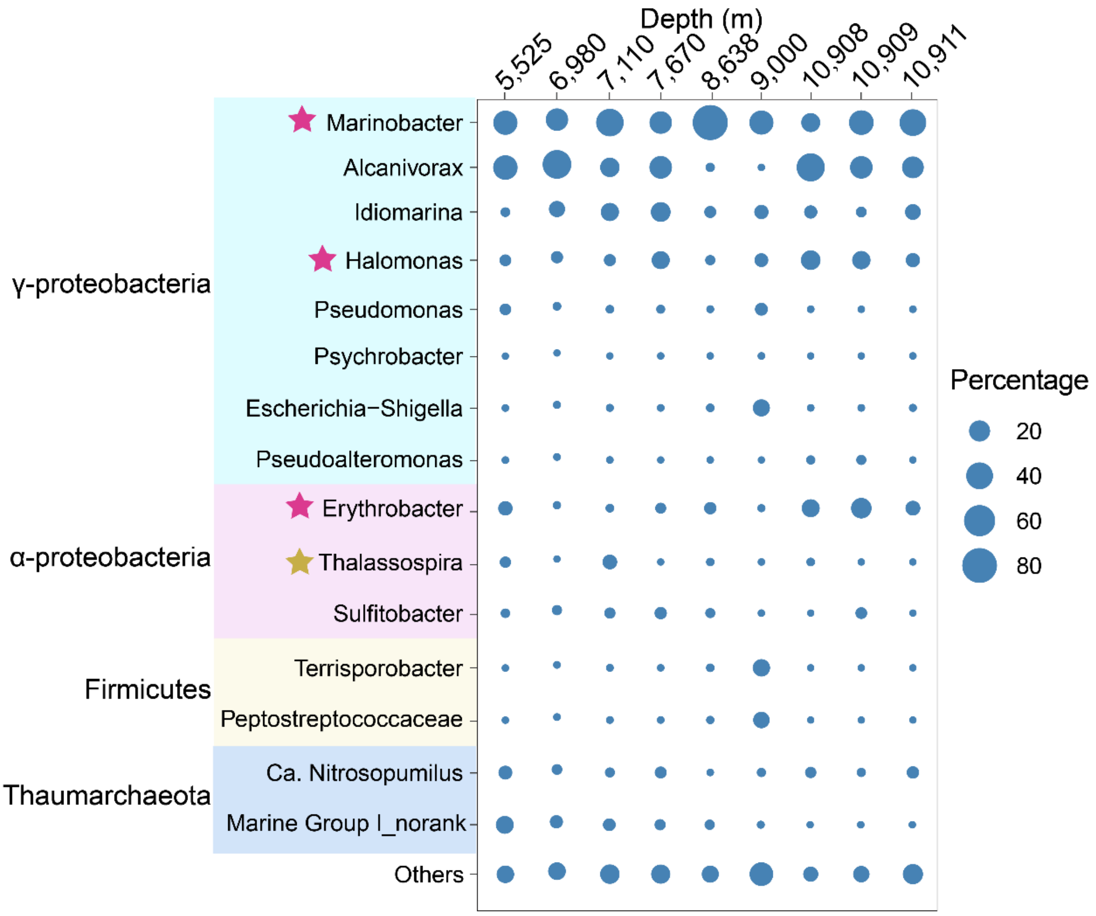

Supplementary Figure 7. Bubble plot depicting the top 15 most abundant genera in sediments at different depths, determined by 16S rRNA gene amplicon sequencing. Stars are coloured to indicate those genera that produce DMSP with unknown DMSP synthesis genes (pink) and *mmtN* (gold).

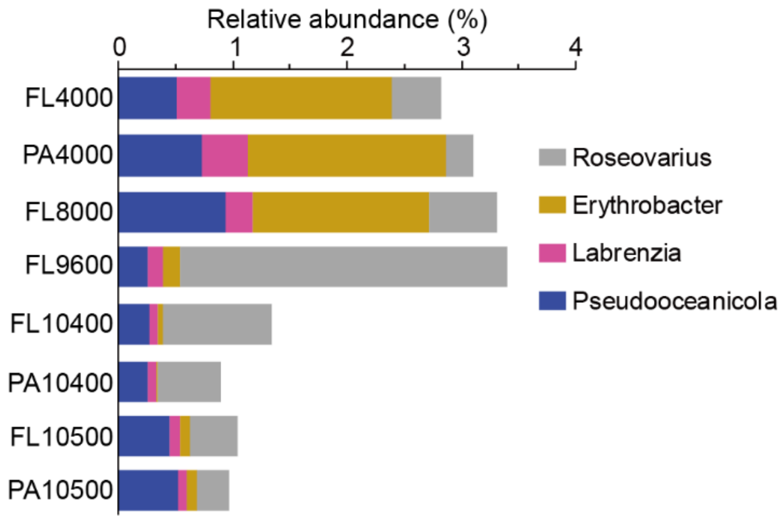

Supplementary Figure 8. The relative abundance of bacterial isolates shown to produce DMSP under physiologically relevant conditions (4°C and 60 MPa). These abundances were determined from metagenome data, i.e. reads number taxonomically assigned to these genera divided by total reads number.

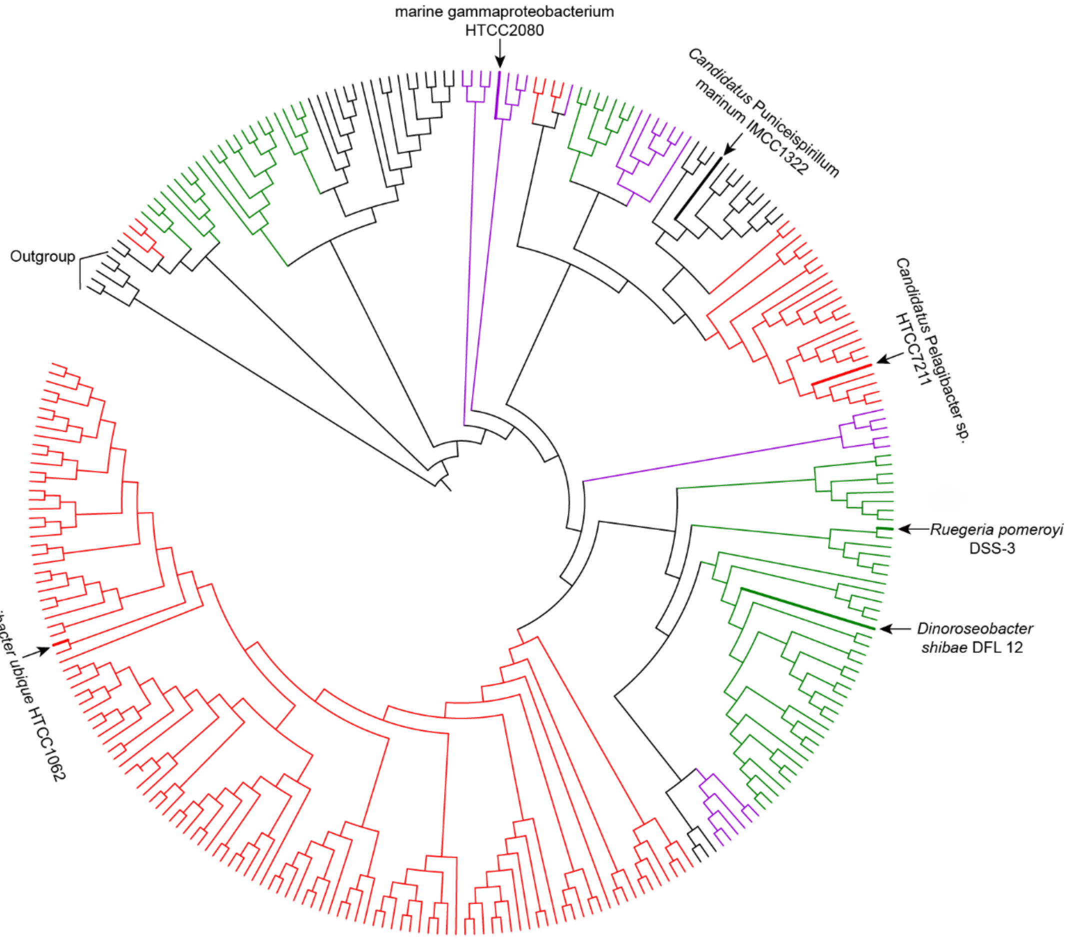

Supplementary Figure 9. Neighbour-joining phylogenetic tree of DmdA proteins predicted from metagenomic data. This tree was constructed from 285 sequences. Sequences experimentally confirmed to demethylate DMSP were used as reference sequences and are labeled in the tree, and the other sequences are from this study. Non-functional DmdA-like proteins (AAV94935, AAV97443, AAZ21486, AAV97197, AAZ22069) were used as the outgroup. Branches in different color indicate different groups. Red: *Pelagibacterales*; Green: *Rhodobacterales*; Purple: *Gammaproteobacteria*; Black: Others.

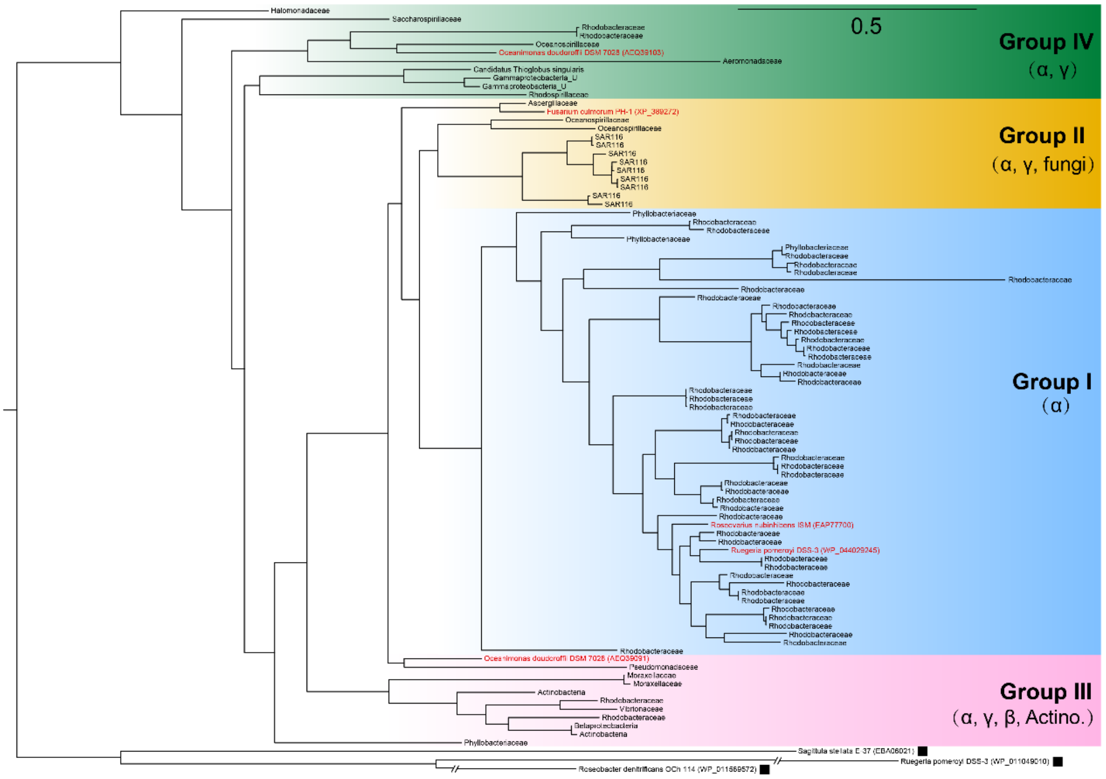

Supplementary Figure 10. Neighbour-joining phylogenetic tree of DddP proteins predicted from metagenomic data. This tree was constructed from 91 sequences. DddP sequences experimentally confirmed to cleave DMSP were used as reference sequences and are labeled in red, and the other sequences are from this study. Non-functional DddP-like protein were used as the outgroup and labeled with a black square. Branches shaded in different colors indicate different groups.

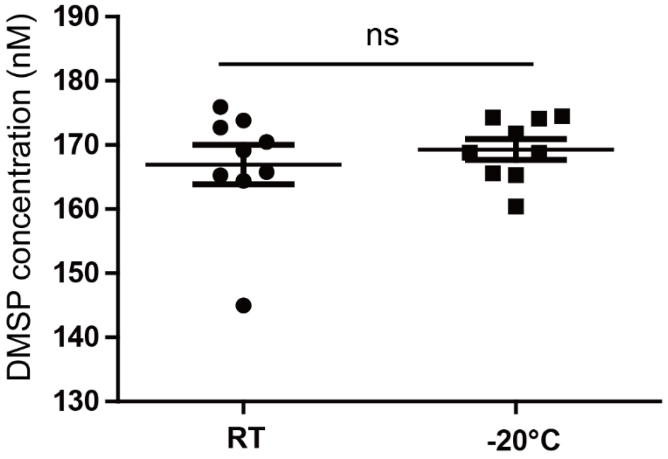

Supplementary Figure 11. Demonstrating that the freezing process did not lead to an overestimation of DMSP. Due to our non-standard procedure of sample storage, we tested whether freezing DMSP and DMS samples in seawater with H_2SO_4 overestimates DMSP due to the inhibition of DMS oxidation. Fresh seawater samples were stored at room temperature and -20°C individually. RT, room temperature; ns, no significant. Data are presented as means ± S.D.

Supplementary Table 1. Characteristics of the sampling sites, including total DMSP concentration in sediment and the seawater column. Temperature and DO concentrations were from Liu et al.[4]. DMSP concentration in ~6,500 m water was from Zhang *et al* [5]. ND, no data. DMSP data are means ± S.D.

Samples	Sampling date	Depth (m)	Location (Lat., Long.)	DMSP (nmol ml^{-1} or g^{-1})	DMS (nmol ml^{-1})	Chl-*a* (μg L^{-1})	Salinity (PSU)	Temp. (°C)	Pressure (~MPa)
Seawater	03.2017	0	11°18.000'N, 142°04.020'E	4.20 ± 0.60×10^{-3}	2.49×10^{-3}	0.015	34.06	29.82	0.1
	03.2017	50	11°18.000'N, 142°04.020'E	6.05 ± 0.57×10^{-3}	2.08×10^{-3}	0.033	34.06	29.82	0.5
	03.2017	100	11°18.000'N, 142°04.020'E	6.00 ± 1.54×10^{-3}	3.07×10^{-3}	0.059	34.05	28.62	1.0
	03.2017	120	11°18.000'N, 142°04.020'E	10.51 ± 1.24×10^{-3}	4.18×10^{-3}	0.180	34.80	26.00	1.2
	03.2017	150	11°18.000'N, 142°04.020'E	9.80 ± 1.57×10^{-3}	4.97×10^{-3}	0.053	34.47	12.95	1.5
	03.2017	200	11°18.000'N, 142°04.020'E	4.69 ± 0.87×10^{-3}	3.05×10^{-3}	0.041	34.83	18.85	2.0
	09.2016	500	11°20.605'N, 142°19.557'E	2.39 ± 0.13×10^{-3}	1.06×10^{-3}	0.010	34.50	7.60	5.0
	09.2016	1,000	11°20.605'N, 142°19.557'E	1.76 ± 0.47×10^{-3}	1.6×10^{-4}	0.010	34.53	4.80	10
	09.2016	1,500	11°20.605'N, 142°19.557'E	1.78 ± 0.13×10^{-3}	1.5×10^{-4}	0.010	34.51	3.05	15
	09.2016	2,000	11°20.605'N, 142°19.557'E	1.86 ± 0.71×10^{-3}	1.5×10^{-4}	0.010	34.62	2.31	20
	09.2016	3,000	11°20.605'N, 142°19.557'E	1.49 ± 0.09×10^{-3}	1.5×10^{-4}	0.010	34.66	1.69	30
	09.2016	4,000	11°20.605'N, 142°19.557'E	0.96 ± 0.22×10^{-3}	1.5×10^{-4}	0.010	34.68	1.51	40
	03.2017	6,050	11°18.000'N, 142°04.020'E	ND	2.0×10^{-4}	0.010	34.71	1.61	60
	05.2016	6,500	9°51.850'N, 138°29.750'E	1.34 ± 0.74×10^{-3}	ND	ND	ND	ND	65
	03.2017	8,320	11°18.000'N, 142°04.020'E	ND	1.5×10^{-4}	0.010	34.71	1.96	83
	09.2016	10,400	11°20.605'N, 142°19.557'E	1.10 ± 0.27×10^{-3}	ND	ND	ND	ND	104

Sediment	07.2016	5,525	10°51.360'N, 141°58.500'E	6.14 ± 0.59
	07.2016	6,980	10°59.380'N, 141°57.870'E	5.16 ± 0.12
	07.2016	7,110	10°59.653'N, 141°59.646'E	4.04 ± 0.34
	07.2016	7,670	11°33.189'N, 141°52.380'E	3.15 ± 0.12
	07.2016	8,638	11°11.699'N, 141°48.701'E	3.45 ± 0.03
	03.2017	10,908	11°19.496'N, 142°11.429'E	3.84 ± 0.03
	03.2017	10,909	11°19.678'N, 142°11.587'E	4.42 ± 0.61
	03.2017	10,911	11°19.513'N, 142°11.291'E	4.52 ± 0.03

Supplementary Table 2. qPCR results of water and sediment samples. ND: not detectable. Copies in water samples are the summation of free-living and particle-associated bacteria. Since there is no available metagenome data for sediment, the estimated percentages of each gene are calculated using the averaged 16S rRNA gene copy in one bacterium (i.e. dividing by 3.61[6]), which are shown inside the brackets.

Samples	Depth (m)	16S rRNA gene (copies/L or /g)	*dsyB* (copies/L or /g)	*mmtN* (copies/L or /g)	*dmdA* (copies/L or /g)	*dddP* (copies/L or /g)
**Water**	0	9.73E+07	2.61E+05	ND	2.22E+07	3.51E+05
	1,000	1.01E+06	3.46E+04	ND	2.29E+05	3.22E+03
	2,000	6.26E+06	1.06E+05	ND	3.16E+05	7.61E+02
	3,000	8.34E+06	1.91E+05	ND	5.70E+05	5.19E+04
	4,000	7.68E+06	3.18E+05	ND	3.18E+04	1.21E+04
	8,000	1.11E+07	9.84E+04	ND	4.00E+04	6.27E+02
	9,600	5.92E+07	9.67E+05	ND	3.99E+05	1.42E+04
	10,400	6.31E+07	2.26E+06	ND	9.39E+04	2.22E+03
	10,500	1.15E+08	3.95E+06	ND	1.43E+05	4.17E+03
**Sediment**	5,525	1.04E+08	4.69E+04 (0.16)	1.05E+02 (0.000)	8.30E+01 (0.000)	ND
	6,980	1.23E+08	6.50E+04 (0.19)	4.07E+02 (0.001)	1.75E+02 (0.001)	ND
	7,110	3.62E+07	9.44E+02 (0.01)	1.33E+02 (0.001)	2.18E+03 (0.022)	ND
	7,670	6.77E+07	7.87E+04 (0.42)	6.26E+01 (0.000)	4.62E+02 (0.002)	ND

	8,638	2.07E+07	1.05E+03 (0.02)	ND	4.76E+02 (0.008)	ND
	10,908	1.28E+08	7.00E+04 (0.20)	2.85E+01 (0.000)	1.18E+03 (0.003)	ND
	10,909	1.03E+08	1.08E+05 (0.38)	ND	9.13E+02 (0.003)	ND
	10,911	5.06E+07	4.20E+04 (0.30)	1.46E+02 (0.001)	5.79E+02 (0.004)	ND

Supplementary Table 3. DMSP producing bacteria isolated from seawater. DMSP production was tested under two conditions (with/without Met). The presence of *dsyB* and *mmtN* was tested using *dsyB* and *mmtN* degenerate primers. Bold text indicates isolates potentially containing novel DMSP producing genes. DMSP concentration is reported as nmol DMSP per ml of culture. NT, not tested. Data presented as means ± S.D.

Class	Strain No.	Species	DMSP with Met (nmol/ml)	DMSP without Met (nmol/ml)	Depth (m)	*dsyB* present	*mmtN* present
α-proteobacteria	**EZ43**	***Hyphomonas atlantica***	**7.21±3.69**	**0**	**0**		
	ZXX238	*Maritimibacter alkaliphilus*	25.09±1.15	3.07±0.37	7,500	√	
	EF3	*Maritimibacter alkaliphilus*	3.98±1.05	NT	4,000	√	
	ZXX190	*Paracoccus oceanense*	20.21±3.0	0	8,000	√	
	ZYF642	*Pelagibaca bermudensis*	12.57±0.60	5.01±1.26	9,600	√	
	RF3	*Ponticoccus litoralis*	62.35±12.05	1.20±0.10	4,000	√	
	ZXX049	*Pseudooceanicola nanhaiensis*	40.0±2.08	33.75±5.16	0	√	
	ZYF240	*Pseudooceanicola nanhaiensis*	16.13±0.91	20.10±3.54	8,000	√	
	ZYF258	*Pseudooceanicola nanhaiensis*	62.50±20.0	16.52±2.74	8,000	√	
	ZXX212	*Sagittula stellata*	8.7±3.03	1.43±0.96	7,500	√	
	EO36	*Sagittula stellata*	7.57±0.75	1.87±0.08	1,000	√	
	**ZXX143**	***Altererythrobacter xiamenensis***	**7.95±0.43**	**0**	**7,500**		
	ZYF522	*Roseovarius indicus*	43.78±2.67	6.51±1.87	10,400	√	√
	**RF22**	***Erythrobacter flavus***	**14.28±0.25**	**0.97±0.17**	**4,000**		
	**RO71**	***Erythrobacter vulgaris***	**12.92±0.33**	NT	**1,000**		

	**RT29**	***Erythrobacter citreus***	**6.77±1.77**	**NT**	**2,000**	
	**ZYF708**	***Erythrobacter citreus***	**47.72±2.40**	**NT**	**9,600**	
	RF14	*Labrenzia aggregata*	40.91±2.19	10.29±4.99	4,000	√
	ZYF703	*Labrenzia aggregata*	56.58±4.78	6.62±1.66	9,600	√
	ZYF612	*Labrenzia aggregata*	392.89±158.86	45.64±24.20	9,600	√
	ZYF403	*Labrenzia aggregata*	68.12±1.80	5.56±1.14	10,400	√
	**ZXX173**	***Marinobacter vinifirmus***	**8.1±0.27**	**0**	**8,000**	
	**ZXX189**	***Luteimonas terrae***	**7.13±0.31**	**0**	**8,000**	
γ-proteobacteria	**RT37**	***Halomonas saccharevitans***	**7.85±0.67**	**NT**	**2,000**	
	**EF61**	***Halomonas alimentaria***	**39.03±0.32**	**1.16±0.25**	**4,000**	
	**ZXX174**	***Halomonas saccharevitans***	**4.27±0.83**	**NT**	**8,000**	
Actinobacteria	**ZYF656**	***Mycobacterium poriferae***	**17.61±0.10**	**0**	**9,600**	

Supplementary Table 4. The capacity of isolates to produce DMSP under physiologically relevant conditions. DMSP concentration is reported as nmol DMSP per ml of culture. Data are presented as means ± S.D.

Strain no.	Top-hit taxon	Similarity (%)	Depth (m)	DMSP without Met (nmol/ml)
ZYF258	*Pseudooceanicola nanhaiensis*	100	8,000	2.61±0.04
ZYF522	*Roseovarius indicus*	100	10,400	2.36±0.21
RF14	*Labrenzia aggregata*	100	4,000	3.40±0.09
ZYF703		100	9,600	2.63±0.05
ZYF612		100	9,600	3.00±0.29
ZYF403		100	10,400	2.67±0.07
ZYF708	*Erythrobacter citreus*	99.01	9,600	1.35±0.11

Supplementary Table 5. The functional and non-functional reference sequences used in this study.

Gene names	Experimentally verified	Microorganisms	Accession number
**DSYB**	Functional	*Prymnesium parvum* CCAP946/6	-
		Chrysochromulina tobin CCMP291	KOO32714
		Lingulodinium polyedrum CCMP1936	-
		Alexandrium tamarense ATSP1-B	-
		Acropora cervicornis	-
		Fragilariopsis cylindrus CCMP1102	OEU17621
		Symbiodinium microadriaticum CCMP2467	OLQ07620
**TpMMT**	Functional	*Thalassiosira pseudonana* CCMP1335	Tp23128
**DsyB**	Functional	*Labrenzia aggregata* IAM 12614	WP_006937642
		Labrenzia aggregate LZB033	WP_075282486
		Pseduooceanicola batsensis HTCC2597	WP_009805585
		Pelagibaca bermudensis HTCC2601	WP_007801186
		Sediminimonas qiaohouensis DSM 21189	WP_026756701
		Thalassobaculum salexigens DSM 19539	WP_084618911
		Sagittula stellate E-37	WP_005854984
		Amorphus coralli DSM 19760	WP_018697905
	Non-functional	*Bacillus mycoides* DSM 2048	EEL96501
		Streptomyces varsoviensis DSM40346	WP_030879264
**MmtN**	Functional	*Novosphingobium* sp. MBES04	WP_052321947
		Croceicoccus mobilis	WP_066775518
		Thalassospira sp. HJ	WP_044830103
		Thalassospira sp. MCCC_1A01148	WP_062957385
		Thalassospira indica	WP_064788038

		Thalassospira tepidiphila MCCC_1A03514	WP_064780488
		Thalassospira australica	WP_033070178
		Thalassospira lucentensis	WP_022734010
		Thalassospira sp. MCCC_1A02898	WP_063085993
		Thalassospira profundimaris sp. DSM17430	WP_008888945
		Labrenzia sp. OB1	WP_068409229
		Roseovarius indicus 01	WP_064261696
		Roseovarius indicus 02	WP_057814729
		Roseovarius indicus 03	KRS18724
		Rhodobacter aestuarii	WP_076485456
		Saccharothrix syringae	WP_033429235
		Micromonospora nigra	WP_091090849
		Agrobacterium vitis	WP_071204336
		Nocardiopsis chromatogenes	WP_017624909
		Streptomyces mobaraensis NBRC_13819	EME99407
**DmdA**	Functional	*Ruegeria pomeroyi* DSS-3	AAV95190
		Pelagibacter ubique HTCC1062	WP_011281570
		Dinoroseobacter shibae DFL 12	WP_012178987
		marine gammaproteobacterium HTCC2080	WP_007233625
		Candidatus Pelagibacter sp. HTCC7211	WP_008546106
		Candidatus Puniceispirillum marinum IMCC1322	WP_013044947
	Non-functional	*Ruegeria pomeroyi* DSS-3	AAV94935
		Ruegeria pomeroyi DSS-3	AAV97443
		Candidatus Pelagibacter ubique HTCC1062	AAZ21486
		Ruegeria pomeroyi DSS-3	AAV97197
		Candidatus Pelagibacter ubique HTCC1062	AAZ22069

		Haloarcula marismortui ATCC 43049	AAV45054
**DddD**	Functional	*Marinomonas* sp. MWYL1	ABR72937
		Oceanimonas doudoroffii DSM 7028	AEQ39135
		Psychrobacter sp. J466	ACY02894
		Halomonas sp. HTNK1	ACV84065
		Burkholderia ambifaria AMMD	WP_011659284
		Pseudomonas sp. J465	ACY01992
	Non-functional	*Ruegeria pomeroyi* DSS-3	AAV94987
		Dinoroseobacter shibae DFL12	ABV95365
		Streptosporangium roseum DSM 43021	ACZ87836
		Emiliania huxleyi CCMP1516	EOD33233
**DddL**	Functional	*Sulfitobacter* sp. EE-36	ADK55772
		Rhodobacter_sphaeroides	WP_011336734
		Rhodobacter sphaeroides 2.4.1	YP_351475
		Fulvimarina_pelagi	WP_007067665
		Loktanella_vestfoldensis	WP_019955302
		Pseudooceanicola_batsensis	WP_009805827
		Labrenzia aggregata LZB033	AKS25183
		*Labrenzia_aggregata*_IAM12614	KP639183
**DddP**	Functional	*Roseovarius nubinhibens* ISM	EAP77700
		Ruegeria pomeroyi DSS-3	WP_044029245
		Oceanimonas doudoroffii DSM 7028	AEQ39091
		Oceanimonas doudoroffii DSM 7028	AEQ39103
		Fusarium graminearum PH-1	XP_389272
	Non-functional	*Ruegeria pomeroyi* DSS-3	WP_011049010
		Sagittula stellata E-37	EBA06021

		Roseobacter denitrificans OCh 114	WP_011569572
**DddQ**	Functional	*Ruegeria pomeroyi* DSS-3	WP_011047333
		Roseovarius nubinhibens ISM	EAP76002
		Roseovarius nubinhibens ISM	EAP76001
		Ruegeria lacuscaerulensis ITI-1157	WP_005978225
		GOS databases	ECW91654
		GOS databases	EBP74803
		GOS databases	ECX82089
**DddW**	Functional	*Ruegeria pomeroyi* DSS-3	AAV93771
**DddY**	Functional	*Alcaligenes faecalis* M3A	ADT64689
		Shewanella putrefaciens CN-32	ABP77243
		Desulfovibrio acrylicus	SHJ73420
		Ferrimonas kyonanensis DSM 18153	WP_028114584
		Acinetobacter bereziniae	ENV21217
**DddK**	Functional	*Candidatus* Pelagibacter ubique HTCC1062	AAZ21215
		Alphaproteobacterium HIMB5	AFS47241
		Candidatus Pelagibacter ubique HTCC9022	WP_028037226
	Non-functional	Alphaproteobacterium HIMB114	EMH79844
		Candidatus Pelagibacter sp. IMCC9063	AEA81283

Supplementary Table 6. Bacterial isolates from seawater with DMSP lyase activity. All strains listed produced 3.82 - 555.63 μM DMS from the 1 mM DMSP added in 24 hours.

Class	Strain No.	Species	Depth (m)
Actinobacteria	ZXX112	*Brevibacterium casei*	8,000
Bacteroidetes	ZXX205-1	*Winogradskyella flava*	7,500
	ZXX215	*Winogradskyella echinorum*	7,500
Firmicutes	ZXX038	*Bacillus flexus*	4,000
	ZXX042	*Bacillus hwajinpoensis*	4,000
	ZXX081-1	*Bacillus horikoshii*	6,000
	ZXX164	*Bacillus vietnamensis*	6,000
	ZXX102	*Bacillus infantis*	7,500
	ZXX103	*Bacillus marisflavi*	7,500
	ZXX151	*Oceanobacillus kapialis*	7,500
	ZXX193	*Bacillus kochii*	8,000
α-proteobacteria	EF1	*Marinovum algicola*	4000
	ZXX271-1	*Marinovum algicola*	7,500
	EF3	*Maritimibacter alkaliphilus*	4,000
	EZ13	*Nautella italica*	0
	ZXX272	*Paracoccus homiensis*	7,500
	ZXX049	*Pseudooceanicola nanhaiensis*	0
	EF2	*Roseovarius pacificus*	4,000
	RN19	*Ruegeria mobilis*	9,000
	ZXX027-1	*Ruegeria mobilis*	0
	EO36	*Sagittula stellata*	1000
	RZ38	*Thalassococcus lentus*	0
	RT29	*Erythrobacter citreus*	10,000
	RF14	*Labrenzia aggregata*	4,000
	ZYF703	*Labrenzia aggregata*	9,600
	ZYF612	*Labrenzia aggregata*	9,600
	ZYF403	*Labrenzia aggregata*	10,400
γ-proteobacteria	ZXX135	*Alteromonas macleodii*	7,500
	ZXX119	*Idiomarina fontislapidosi*	8,000
	ES9	*Marinobacter flavimaris*	6,000

ZXX279	*Marinobacter salsuginis*	7,500
ES2	*Pseudoalteromonas shioyasakiensis*	6,000
ZXX053	*Pseudoalteromonas arabiensis*	0
ZXX094	*Pseudomonas stutzeri*	8,000
ZXX240	*Halomonas meridiana*	8,000
EF61	*Halomonas alimentaria*	4,000
RT37	*Halomonas saccharevitans*	2,000
ZXX166	*Halomonas venusta*	6,000
ZXX098	*Salinicola salarius*	7,500
ZXX278	*Psychrobacter faecalis*	7,500
ZXX021	*Vibrio antiquarius*	200
ZXX051	*Vibrio alginolyticus*	0
ZXX011	*Vibrio neocaledonicus*	200

Supplementary Table 7. Primers used in this study. The bases underlined with wavy lines are reverse complementary regions for overlap PCR. The bases that are underlined are restriction enzyme sites.

Gene name	Primer sequences (5' - 3')	Annealing temp. (°C)	Length (bp)	Function and reference
16S rRNA gene	515F: GTGYCAGCMGCCGCGGTAA<br>806R: GGACTACNVGGGTWTCTAAT	55	291	Amplicon sequencing for sediments[7]
16S rRNA gene	Eub338F: ACTCCTACGGGAGGCAGCAG<br>Eub518R: ATTACCGCGGCTGCTGG	55	180	qPCR[8]
dsyB	dsyB_deg1F: CATGGGSTCSAAGGCSCTKTT<br>dsyB_deg2R: GCAGRTARTCGCCGAAATCGTA	58	246	qPCR and RT-qPCR[3]
mmtN	mmtN_degF: CCGAGGTGGTCATGAAYTTYGG<br>mmtN_degR: GGATCACGCACACYTCRTGRTA	54	301	qPCR[3]
dddP	dddP_874F: AAYGAAATWGTTGCCTTTGA<br>dddP_971R: GCATDGCRTAAATCATATC	44	97	qPCR[8]
dmdA subclade A/1	A/1-spFP: ATGGTGATTTGCTTCAGTTTCT<br>A/1-spRP: CCCTGCTTTGACCAACC	53	228	qPCR[9]
dmdA subclade A/2	A/2-spFP: CGATGAACATTGGTGGGTTTCTA<br>A/2-spRP: GCCATTAGGTCGTCTGATTTTGG	59	147	qPCR[9]
dmdA subclade B/3	B/3-spFP: GATGTCTCCTGCCAACGTCAGGTCGA<br>B/3-spRP: ACCGGGTCATTGATCATGCCTGCG	62	154	qPCR[9]
dmdA subclade C/2	C/2-spFP: AGATGAAAATGCTGGAATGATAAATG	50	191	qPCR[9]

	C/2-spRP: AAATCTTCAGACTTTGGACCTTG			
dmdA subclade D/1	D/1-spFP: AGATGTTATTATTGTCCAATAATTGATG<br>D/1-spRP: ATCCACCATCTATCTTCAGCTA	49	89	qPCR[9]
dmdA subclade D/3	D/3-spFP: AATGGTGGATTTCTATTGCAGATAC<br>D/3-spRP: GATTTTGGACCTTGTACAGCCA	54	126	qPCR[9]
dmdA subclade E/2	E/2-spFP: CATGTTCAGATCTGGGACGT<br>E/2-spRP: AGCGGCACATACATGCACT	57	133	qPCR[9]
J526-DdsyB-UO	GCTCTAGACGTGACCTATGCCGACCCGC (XbaI)			UO/UI and DO/DI are for PCR amplification of the upstream and downstream homologous arms of *dsyB*, respectively
J526-DdsyB-UI	CTCGGAGACCGTCACCTGTGACCCCATGAACCC			
J526-DdsyB-DI	TGACGGTCTCCGAGATGATCC			
J526-DdsyB-DO	GCCCAAGCTTGCCGAAGAAAACCATCGATCC (HindIII)			
La 6-DdsyB-UO	CGGAATTCGATGATCGGGCTGGGCGTGG (EcoRI)			Same as above
La 6-DdsyB-UI	CCGATGTCAGCCACATGTTGGAAATCTCGTCGGC			
La 6-DdsyB-DI	GTGGCTGACATCGGAGCTGGAAG			
La 6-DdsyB-DO	GCTCTAGACCGCTGATGTGGGGCGACCTGG (XbaI)			

Supplementary References

1. Zhang, X.H. *et al.* Biogenic production of DMSP and its degradation to DMS—their roles in the global sulfur cycle. *Sci. China Life Sci.* **62**, 1296-1319 (2019).
2. Curson, A.R. *et al.* Dimethylsulfoniopropionate biosynthesis in marine bacteria and identification of the key gene in this process. *Nat. Microbiol.* **2**, 17009 (2017).
3. Williams, B.T. *et al.* Bacteria are important dimethylsulfoniopropionate producers in coastal sediments. *Nat. Microbiol.* **4**, 1815-1825 (2019).
4. Liu, J. *et al.* Proliferation of hydrocarbon-degrading microbes at the bottom of the Mariana Trench. *Microbiome* **7**, 47 (2019).
5. Zhang, M., Sun, C., Yang, G. & Ding, H. The vertical variation characteristics of CH4 and DMSP in the seawater of the Yap Trench in the western Pacific Ocean. *Haiyang Xuebao* **40**, 143-157 (2018). (in Chinese, abstract in English).
6. Sun, D.L., Jiang, X., Wu, Q.L. & Zhou, N.Y. Intragenomic heterogeneity of 16S rRNA genes causes overestimation of prokaryotic diversity. *Appl. Environ. Microbiol.* **79**, 5962-9 (2013).
7. Walters, W. *et al.* Improved bacterial 16S rRNA gene (V4 and V4-5) and fungal internal transcribed spacer marker gene primers for microbial community surveys. *mSystems* **1**, e00009-15 (2016).
8. Yin, Q., Fu, B., Li, B., Shi, X., Inagaki, F. & Zhang, X. Spatial variations in microbial community composition in surface seawater from the ultra-oligotrophic center to rim of the South Pacific Gyre. *PloS ONE* **8,** e55148 (2013).
9. Varaljay, V.A., Howard, E.C., Sun, S.L. & Moran, M.A. Deep Sequencing of a Dimethylsulfoniopropionate-Degrading Gene (dmdA) by Using PCR Primer Pairs Designed on the Basis of Marine Metagenomic Data. *App. Environ. Microbiol.* **76**, 609-617 (2010).
