# Supplementary material for: Bacteria are important dimethylsulfoniopropionate producers in marine aphotic and high-pressure environments
Source: Nat Commun. 2020 Sep 16;11:4658. doi: 10.1038/s41467-020-18434-4 (PMC7494906; doi:10.1038/s41467-020-18434-4)
Supplement: Supplementary file 2 — Description of Additional Supplementary Files [file 41467_2020_18434_MOESM2_ESM.docx]

**Supplementary Data 1**. Metagenome assembled genomes (MAGs) from various water depths that harboured DMSP cycling genes.
