# Supplementary material for: Bacteria are important dimethylsulfoniopropionate producers in marine aphotic and high-pressure environments
Source: Nat Commun. 2020 Sep 16;11:4658. doi: 10.1038/s41467-020-18434-4 (PMC7494906; doi:10.1038/s41467-020-18434-4)
Supplement: Supplementary file 4 — Reporting Summary [file 41467_2020_18434_MOESM4_ESM.pdf]

nature research

Corresponding author(s): Prof. Xiao-Hua Zhang and Prof. Jonathan D. Todd

Last updated by author(s): Aug 19, 2020

Reporting Summary

Nature Research wishes to improve the reproducibility of the work that we publish. This form provides structure for consistency and transparency in reporting. For further information on Nature Research policies, see Authors & Referees and the Editorial Policy Checklist.

Statistics

For all statistical analyses, confirm that the following items are present in the figure legend, table legend, main text, or Methods section.

n/a	Confirmed	
☐	☒	The exact sample size (n) for each experimental group/condition, given as a discrete number and unit of measurement
☐	☒	A statement on whether measurements were taken from distinct samples or whether the same sample was measured repeatedly
☐	☒	The statistical test(s) used AND whether they are one- or two-sided<br>*Only common tests should be described solely by name; describe more complex techniques in the Methods section.*
☒	☐	A description of all covariates tested
☐	☒	A description of any assumptions or corrections, such as tests of normality and adjustment for multiple comparisons
☐	☒	A full description of the statistical parameters including central tendency (e.g. means) or other basic estimates (e.g. regression coefficient) AND variation (e.g. standard deviation) or associated estimates of uncertainty (e.g. confidence intervals)
☐	☒	For null hypothesis testing, the test statistic (e.g. F, t, r) with confidence intervals, effect sizes, degrees of freedom and P value noted<br>*Give P values as exact values whenever suitable.*
☒	☐	For Bayesian analysis, information on the choice of priors and Markov chain Monte Carlo settings
☒	☐	For hierarchical and complex designs, identification of the appropriate level for tests and full reporting of outcomes
☒	☐	Estimates of effect sizes (e.g. Cohen's d, Pearson's r), indicating how they were calculated

Our web collection on statistics for biologists contains articles on many of the points above.

Software and code

Policy information about availability of computer code

Data collection	No software was used for data collection
Data analysis	MEGA (v7.0), MegaHit (v1.1.2), MaxBin2 (v2.2.4), metaBAT2 (v2.12.1), CONCOCT (v0.4.0), CheckM, Taxator-tk (v1.3.3e), QIIME (v1.9), ggplot (v2.2.1), HMMER (v3.1)

For manuscripts utilizing custom algorithms or software that are central to the research but not yet described in published literature, software must be made available to editors/reviewers. We strongly encourage code deposition in a community repository (e.g. GitHub). See the Nature Research guidelines for submitting code & software for further information.

Data

Policy information about availability of data

All manuscripts must include a data availability statement. This statement should provide the following information, where applicable:

- Accession codes, unique identifiers, or web links for publicly available datasets
- A list of figures that have associated raw data
- A description of any restrictions on data availability

Sequence data for metagenome of water samples from September 2016 (PRJNA412741) and March 2017 (PRJNA541485), and 16S rRNA reads of water samples (PRJNA413447) and sediment samples (PRJNA416963) have been deposited in the NCBI Sequence Read Archive.

Field-specific reporting

Please select the one below that is the best fit for your research. If you are not sure, read the appropriate sections before making your selection.

☒ Life sciences ☐ Behavioural & social sciences ☐ Ecological, evolutionary & environmental sciences

For a reference copy of the document with all sections, see nature.com/documents/nr-reporting-summary-flat.pdf

Life sciences study design

All studies must disclose on these points even when the disclosure is negative.

Sample size	No sample size calculations were performed. Field experiments in the Mariana trench were done with biological replicates. For metagenomics and 16S rRNA gene amplicon sequencing analysis, replicates (0-8,000 m waters) were sequenced.
Data exclusions	No data exclusion.
Replication	All measurements of DMSP concentration from cultures and environmental samples reported are from biological replicates. Furthermore, the growth of wild and dsyB mutant strains under pressure was reproduced independently.
Randomization	No randomization was done
Blinding	No blinding was done as this is not applicable.

Reporting for specific materials, systems and methods

We require information from authors about some types of materials, experimental systems and methods used in many studies. Here, indicate whether each material, system or method listed is relevant to your study. If you are not sure if a list item applies to your research, read the appropriate section before selecting a response.

Materials & experimental systems

n/a	Involved in the study
☒	☐ Antibodies
☒	☐ Eukaryotic cell lines
☒	☐ Palaeontology
☒	☐ Animals and other organisms
☒	☐ Human research participants
☒	☐ Clinical data

Methods

n/a	Involved in the study
☒	☐ ChIP-seq
☐	☒ Flow cytometry
☒	☐ MRI-based neuroimaging

Flow Cytometry

Plots

Confirm that:

☒ The axis labels state the marker and fluorochrome used (e.g. CD4-FITC).

☒ The axis scales are clearly visible. Include numbers along axes only for bottom left plot of group (a 'group' is an analysis of identical markers).

☒ All plots are contour plots with outliers or pseudocolor plots.

☐ A numerical value for number of cells or percentage (with statistics) is provided.

Methodology

Sample preparation	Seawater samples were fixed with paraformaldehyde (final concentration 1%), incubated for at least 30 min in the dark and then quick frozen in liquid nitrogen until flow cytometric analysis in the laboratory. Before analysis, samples of heterotrophic bacteria were diluted 5-fold with TE buffer, stained with SYBR Green I (Molecular Probes, USA) (final concentration 0.01%) and incubated 20 min in the dark.
Instrument	Samples were analyzed with a BD FACSJazz flow cytometer (Becton Dickinson).
Software	Flow cytometric data were collected and analyzed with BD FACS Sortware and Summit software, respectively.
Cell population abundance	The measured cell abundances varied from 0.1 ×105 to 5.8×105 cells mL-1

Gating strategy

Cells were resolved on the basis of their fluorescence and scatter properties.

☒ Tick this box to confirm that a figure exemplifying the gating strategy is provided in the Supplementary Information.
